# Genomic Evidence for the Nonpathogenic State in HIV-1–Infected Northern Pig-Tailed Macaques

**DOI:** 10.1093/molbev/msad101

**Published:** 2023-05-03

**Authors:** Wei Pang, Yong Shao, Xiao-Lin Zhuang, Ying Lu, Wen-Qiang He, Hong-Yi Zheng, Rong Xin, Ming-Xu Zhang, Xiao-Liang Zhang, Jia-Hao Song, Ren-Rong Tian, Fan Shen, Yi-Hui Li, Zu-Jiang Zhao, Dong-Dong Wu, Yong-Tang Zheng

**Affiliations:** Key Laboratory of Animal Models and Human Disease Mechanisms of the Chinese Academy of Sciences, KIZ-CUHK Joint Laboratory of Bioresources and Molecular Research in Common Diseases, Center for Biosafety Mega-Science, Kunming Institute of Zoology, Chinese Academy of Sciences, Kunming, Yunnan, China; State Key Laboratory of Genetic Resources and Evolution, Kunming Institute of Zoology, Chinese Academy of Sciences, Kunming, China; State Key Laboratory of Genetic Resources and Evolution, Kunming Institute of Zoology, Chinese Academy of Sciences, Kunming, China; Key Laboratory of Animal Models and Human Disease Mechanisms of the Chinese Academy of Sciences, KIZ-CUHK Joint Laboratory of Bioresources and Molecular Research in Common Diseases, Center for Biosafety Mega-Science, Kunming Institute of Zoology, Chinese Academy of Sciences, Kunming, Yunnan, China; Kunming College of Life Science, University of the Chinese Academy of Sciences, Kunming, China; Key Laboratory of Animal Models and Human Disease Mechanisms of the Chinese Academy of Sciences, KIZ-CUHK Joint Laboratory of Bioresources and Molecular Research in Common Diseases, Center for Biosafety Mega-Science, Kunming Institute of Zoology, Chinese Academy of Sciences, Kunming, Yunnan, China; Key Laboratory of Animal Models and Human Disease Mechanisms of the Chinese Academy of Sciences, KIZ-CUHK Joint Laboratory of Bioresources and Molecular Research in Common Diseases, Center for Biosafety Mega-Science, Kunming Institute of Zoology, Chinese Academy of Sciences, Kunming, Yunnan, China; Key Laboratory of Animal Models and Human Disease Mechanisms of the Chinese Academy of Sciences, KIZ-CUHK Joint Laboratory of Bioresources and Molecular Research in Common Diseases, Center for Biosafety Mega-Science, Kunming Institute of Zoology, Chinese Academy of Sciences, Kunming, Yunnan, China; Kunming College of Life Science, University of the Chinese Academy of Sciences, Kunming, China; Key Laboratory of Animal Models and Human Disease Mechanisms of the Chinese Academy of Sciences, KIZ-CUHK Joint Laboratory of Bioresources and Molecular Research in Common Diseases, Center for Biosafety Mega-Science, Kunming Institute of Zoology, Chinese Academy of Sciences, Kunming, Yunnan, China; Key Laboratory of Animal Models and Human Disease Mechanisms of the Chinese Academy of Sciences, KIZ-CUHK Joint Laboratory of Bioresources and Molecular Research in Common Diseases, Center for Biosafety Mega-Science, Kunming Institute of Zoology, Chinese Academy of Sciences, Kunming, Yunnan, China; Key Laboratory of Animal Models and Human Disease Mechanisms of the Chinese Academy of Sciences, KIZ-CUHK Joint Laboratory of Bioresources and Molecular Research in Common Diseases, Center for Biosafety Mega-Science, Kunming Institute of Zoology, Chinese Academy of Sciences, Kunming, Yunnan, China; Key Laboratory of Animal Models and Human Disease Mechanisms of the Chinese Academy of Sciences, KIZ-CUHK Joint Laboratory of Bioresources and Molecular Research in Common Diseases, Center for Biosafety Mega-Science, Kunming Institute of Zoology, Chinese Academy of Sciences, Kunming, Yunnan, China; Key Laboratory of Animal Models and Human Disease Mechanisms of the Chinese Academy of Sciences, KIZ-CUHK Joint Laboratory of Bioresources and Molecular Research in Common Diseases, Center for Biosafety Mega-Science, Kunming Institute of Zoology, Chinese Academy of Sciences, Kunming, Yunnan, China; Key Laboratory of Animal Models and Human Disease Mechanisms of the Chinese Academy of Sciences, KIZ-CUHK Joint Laboratory of Bioresources and Molecular Research in Common Diseases, Center for Biosafety Mega-Science, Kunming Institute of Zoology, Chinese Academy of Sciences, Kunming, Yunnan, China; Key Laboratory of Animal Models and Human Disease Mechanisms of the Chinese Academy of Sciences, KIZ-CUHK Joint Laboratory of Bioresources and Molecular Research in Common Diseases, Center for Biosafety Mega-Science, Kunming Institute of Zoology, Chinese Academy of Sciences, Kunming, Yunnan, China; State Key Laboratory of Genetic Resources and Evolution, Kunming Institute of Zoology, Chinese Academy of Sciences, Kunming, China; Center for Excellence in Animal Evolution and Genetics, Chinese Academy of Sciences, Kunming, Yunnan, China; National Resource Center for Non-Human Primates, Kunming Primate Research Center, and National Research Facility for Phenotypic & Genetic Analysis of Model Animals (Primate Facility), Kunming Institute of Zoology, Chinese Academy of Sciences, Kunming, Yunnan, China; Kunming Natural History Museum of Zoology, Kunming Institute of Zoology, Chinese Academy of Sciences, Kunming, Yunnan, China; Key Laboratory of Animal Models and Human Disease Mechanisms of the Chinese Academy of Sciences, KIZ-CUHK Joint Laboratory of Bioresources and Molecular Research in Common Diseases, Center for Biosafety Mega-Science, Kunming Institute of Zoology, Chinese Academy of Sciences, Kunming, Yunnan, China; Kunming College of Life Science, University of the Chinese Academy of Sciences, Kunming, China; National Resource Center for Non-Human Primates, Kunming Primate Research Center, and National Research Facility for Phenotypic & Genetic Analysis of Model Animals (Primate Facility), Kunming Institute of Zoology, Chinese Academy of Sciences, Kunming, Yunnan, China

**Keywords:** northern pig-tailed macaque, HIV-1, genome, transcriptome, *TLR8*, *IFI27*

## Abstract

HIV-1 is a highly host-specific retrovirus that infects humans but not most nonhuman primates. Thus, the lack of a suitable primate model that can be directly infected with HIV-1 hinders HIV-1/AIDS research. In the previous study, we have found that the northern pig-tailed macaques (NPMs) are susceptible to HIV-1 infection but show a nonpathogenic state. In this study, to understand this macaque–HIV-1 interaction, we assembled a de novo genome and longitudinal transcriptome for this species during the course of HIV-1 infection. Using comparative genomic analysis, a positively selected gene, Toll-like receptor 8, was identified with a weak ability to induce an inflammatory response in this macaque. In addition, an interferon-stimulated gene, interferon alpha inducible protein 27, was upregulated in acute HIV-1 infection and acquired an enhanced ability to inhibit HIV-1 replication compared with its human ortholog. These findings coincide with the observation of persistently downregulated immune activation and low viral replication and can partially explain the AIDS-free state in this macaque following HIV-1 infection. This study identified a number of unexplored host genes that may hamper HIV-1 replication and pathogenicity in NPMs and provided new insights into the host defense mechanisms in cross-species infection of HIV-1. This work will facilitate the adoption of NPM as a feasible animal model for HIV-1/AIDS research.

## Introduction

A suitable nonhuman primate model that can be infected with HIV-1 and develop AIDS would facilitate the evaluation of anti-HIV-1 therapies and vaccines, but such a model is currently lacking. This is partly because HIV-1 is a highly host-specific retrovirus that replicates robustly and causes AIDS only in humans but does not infect other primates in general. Although Chimpanzees can be infected with HIV-1, it is greatly limited to use for the endangered status and extreme expense in animal experiment and hardly developing to AIDS after several years of infection (Nath et al. 2000). At present, the frequently used nonhuman primate models for HIV-1/AIDS research are simian immunodeficiency virus (SIV) or chimeric SIV encoding the HIV-1 envelope or reverse transcriptase (SHIV)-infected macaques. These models display similar viral replication and pathogenicity with those in HIV-1–infected humans (Hatziioannou and Evans 2012). However, because of the large genetic difference between HIV-1 and SIV (their genomes only share about 40–50% sequence identity at the nucleotide level) (Pollom et al. 2013), the anti-HIV-1 drug and vaccine evaluations in these models cannot directly applied to clinical studies.

Pig-tailed macaques (PTMs) are the only type of Old-World monkeys that were found to be susceptible to HIV-1 in the 1990s (Agy et al. 1992), and they were then found to be more amenable to infection by the HIV-1 derivative strain stHIV-1 in the 2000s. However, neither natural infection of these two strains in PTMs led to a pathogenic state (Hatziioannou et al. 2009, 2014; Thippeshappa et al. 2011; Schmidt et al. 2019). We have firstly posited that a fusion gene *TRIM5-cyclophilin A* replaced an important HIV-1 restrict factor *TRIM5α* in PTMs, resulting in the loss of anti-HIV-1 activity of this gene and thus rendering PTMs susceptible to HIV-1 infection (Liao et al. 2007). Thereafter, we found that one species of PTMs, northern pig-tailed macaque (*Macaca leonine,* NPM), which inhabits Southwest China and Southeast Asia, is amenable to HIV-1 infection (Kuang et al. 2009). HIV-1 could replicate at a low level and formed viral reservoirs in vivo during several years of infection and did not induce any diseases in this species (Pang et al. 2017). Whereas an HIV-1 derivative strain stHIV-1sv, in which HIV-1 *vif* gene is replaced by SIV_mac_*vif* to counterpart another important HIV-1 host restrict factor APOBEC3 in NPMs, presents a high level of replication in acute infection, but its replication was still impeded in chronic infection and also did not cause AIDS in NPMs (Pang et al. 2018). In stark contrast, SIV infection in this monkey caused a consistently high viremia with a slow AIDS progression (Zhang et al. 2017, 2018). Therefore, it remains unclear why HIV-1, and in particular stHIV-1sv, maintain low levels of replications and do not induce AIDS in NPM compared with SIV.

To gain insight into the underlying molecular mechanism responsible for the distinct outcomes of viral infections, and ultimately, the nonpathogenic state of HIV-1 infection in NPMs, here, we conducted a comparative study between HIV-1 and stHIV-1sv infections in NPMs and SIV infection in NPMs or rhesus macaques (RMs). We assembled the genome of NPM (*M. leonina*) and profiled longitudinal transcriptomes after HIV-1 and SIV infections in NPMs by deep RNA-sequencing. Using comparative genomic and functional analyses, we identified several specific genes that are associated with the AIDS-free state in HIV-1-infected NPMs. In particular, Toll-like receptor 8 (*TLR8*) showed a weak ability to induce an inflammatory response, and interferon alpha inducible protein 27 (*IFI27*) obtained an enhanced capacity of inhibition HIV-1 replication in NPMs. This study provides a comprehensive view of the genomic landscape and transcriptomic changes during HIV-1 infection in nonhuman primates, and facilitates the understanding of the complexity of HIV-1 adaptation and pathogenicity in this monkey population.

## Results

### HIV-1-Infected NPM Exhibited a Nonpathogenic State With Low Viramia and Immune Activation

**Fig. 1.**
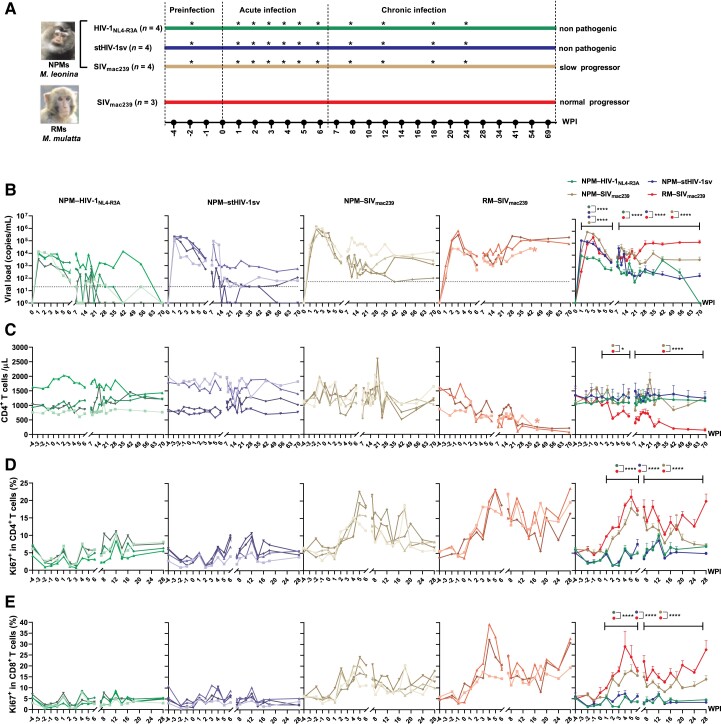
Main virological and immunological characteristics of NPMs infected with HIV-1_NL4-R3*A*_, stHIV-1sv, or SIV_mac239_. (*A*) Twelve healthy NPMs were equally divided into three groups for HIV-1_NL4-R3*A*_, stHIV-1sv, and SIV_mac239_ infection, and three healthy RMs were used for SIV_mac239_ infection. The infection stage was divided into acute infection (0–6 wpi) and chronic infection (7–69 wpi). Asterisks represent time points at which periphPBMCs were collected for transcriptome analysis. Changes in plasma viral load (*B*), CD4^+^ T cells (*C*), CD4^+^ Ki67^+^ T cells (*D*), and CD8^+^ Ki67^+^ T cells (*E*) following HIV-1_NL4-R3*A*_, stHIV-1sv, and SIV_mac239_ infection in NPMs, and SIV_mac239_ infection in RMs. In (*B*), dashed lines mean the limit of quantification, In (*B*) and (*C*), asterisks in RM–SIV_mac239_ panels indicate one RM died of AIDS at 41 wpi. In (*B*)–(*E*), each shape in different infections represents each macaque. Data in the last panel of (*B*)–(*E*) are presented as mean ± SEM, and the data in each infection during 1–6 wpi or 7–69/7–28 wpi are compared with each other by a two-way ANOVA test (*P < 0.05, **P < 0.01, ***P < 0.001, ****P < 0.0001).

We inoculated four NPMs with an HIV-1 strains, HIV-1_NL4-R3A_ (Nolan et al. 2009), and four NPMs with the HIV-1 derivative stHIV-1sv (Hatziioannou et al. 2009), respectively; we also infected four NPMs and three RMs with the SIV strain SIV_mac239_ as positive controls, respectively (fig. 1*A*). The main viral and immunological features during infection were measured. The HIV-1 and SIV infections in NPMs and RMs were divided into acute infection (0–6 weeks post infection [wpi]) and chronic infection (7–69 wpi) according to the setpoints of plasma viral loads. In contrast to the efficient replication of SIV_mac239_ in NPMs and RMs, the replication of HIV-1_NL4-R3A_ was less efficient in NPMs, and stHIV-1sv showed a higher level of acute viremia than that of HIV-1_NL4-R3A_, possibly due to the replacement of SIV *vif*; but its replication was still lower than that of SIV_mac239_ infection in NPMs. However, the replications of both HIV-1 strains were impeded during chronic stage, which kept a relatively low level, approximate 10- to 100-fold reductions than those of SIV-infected NPMs and RMs (fig. 1*B*). The NPMs maintained CD4^+^ T-, CD8^+^ T-, and B-cell homeostasis during HIV-1 and SIV infections (fig. 1*C*; supplementary fig. S1, Supplementary Material online), whereas RMs infected with SIV displayed typical AIDS-defining conditions, including a decrease in CD4^+^ T cells during acute infection and a persistent decline during chronic infection (fig. 1*C*). These results are in line with our previous studies (Pang et al. 2017, 2018; Zhang et al. 2018) and suggest that beside TRIM5α and APOBEC3, some other restrict factors may inhibit HIV-1 replication in NPMs. An indirect indication was stHIV-1sv adaptation to another species of PTMs, southern PTMs, wherein the amino acid changes in viral Vpu, and Gag-capsid proteins have obtained enhanced antagonism to macaque restrict factors tetherin, and MX2, respectively, but the viral swarms or clones did not replicate well and induce the macaques to AIDS states (Hatziioannou et al. 2014; Schmidt et al. 2019), implying that some undiscovered host factors still hamper HIV-1 replication and pathogenicity in that species.

We also detected T-cell activation and proliferation in peripheral blood mononuclear cells (PBMCs) by the changes of the Ki67 marker and found that HIV-1_NL4-R3A_- and stHIV-1sv- infected NPMs exhibited a substantially low level of Ki67^+^ expression on CD4^+^ T and CD8^+^ T cells during the acute and chronic stage, whereas SIV_mac239_-infected NPMs showed a transient and moderate increase of Ki67^+^ expression on CD4^+^ T and CD8^+^ T cells in the acute infection and a decline in the chronic stage. In contrast, SIV_mac239_ infection in RMs presented a robust expression of Ki67 in the whole process (fig. 1*D* and *E*). This feature was consistent with two other immune activation markers, CD38 and HLA-DR (supplementary fig. S2, Supplementary Material online). During the 69-week surveillance period of the infection, the HIV-1− and SIV-infected NPMs did not show any obviously AIDS-related diseases. In contrast, the SIV-infected RMs presented the typical AIDS- defining illnesses, such as the loss of weight and diarrhea (data not shown). Thus, these data demonstrated that: 1) Compared with SIV infections in NPMs and RMs, HIV-1 infections in NPMs presented a lower level of viral replication, especially in chronic infection stage. 2) Compared with SIV infection in RMs, HIV-1 and SIV infections in NPMs led to a weaker level of immune activation. Together, the HIV-1-infected NPMs in particular exhibited a nonpathogenic state with two main features: low viramia and low immune activation.

### Comparative Genomic Analyses Identified Rapidly Evolving Genes in the NPM

To reveal the potential molecular mechanisms underlying the nonpathogenic state in HIV-1-infected NPMs, we assembled the de novo genome of a male NPM, utilizing 425.38 Gb of data sequenced on the Illumina HiSeq X platform from six paired-end libraries constructed with diverse insert sizes **(**supplementary table S1, Supplementary Material online**)**. The scaffold and contig N50 sizes of the draft genome were 5.20 MB and 50.06 KB, respectively **(**supplementary table S2, Supplementary Material online**)**. The genome size of the assembly was 2.88 Gb. By integrating different methods, we annotated 23,570 protein-coding genes **(**supplementary table S3, Supplementary Material online**)**. BUSCO analyses indicated that 89% of 843 single-copy orthologous BUSCO genes were identified in the assembled genome **(**supplementary table S4, Supplementary Material online**)**, supporting the completeness of this assembly. These data demonstrated that the *M. leonina* reference genome was of sufficient quality for further population-scale genome and transcriptome analyses (table 1).

**Table 1.** The Northern Pig-Tailed Macaques (*Macaca leonina*) Genome Assembly Statistics.

Assembly		Annotation	
Average coverage per base	141.79 X	Protein-coding genes	23,570
Total assembly length	2.41 GB	miRNA	17,611
Total sequence length	2.88 GB	tRNA	470
Number of scaffolds	177,242	rRNA	653
Scaffold N50	5,199,335 bp	snRNA	3,379
Number of contigs	275,818		
Contig N50	50,058 bp		
GC content	40.91%		

**Fig. 2.**
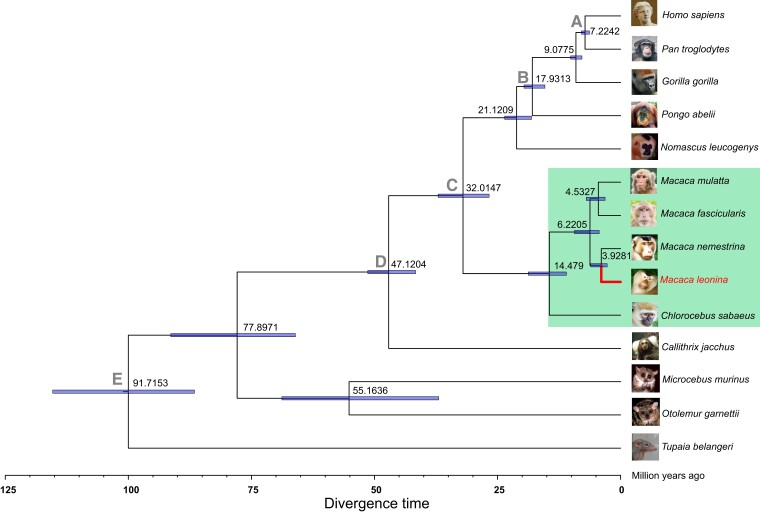
Evolutionary status of the NPM among some representative primates. Consensus phylogenetic tree of 14 species included human being (*Homo sapiens*); 4 apes: chimpanzee (*Pan troglodytes*), western gorilla (*Gorilla gorilla*), sumatran orangutan (*Pongo abeliii*), and northern white-cheeked gibbon (*Nomascus leucogenys*); 5 Old-World monkeys: RMs (*Macaca mulatta*), cynomolgus macaque (*M. fascicularis*), sourthern PTMs (*M. nemestrina*), NPM (*M. leonina*) and African vervet monkey (*Chlorocebus sabaeus*); 3 New-World monkeys: white-tufted-ear marmoset (*Callithrix jacchus*), small-eared galago (*Otolemur garnettii*), gray mouse lemur (*Microcebus murinus*); and an experimental animal close affinity to primate: Chinese tree shrew (*Tupaia belangeri*). The MCMCTree algorithm in PAML4 (Yang 2007) was utilized to evaluate divergence time among 14 species. The nodal ages and the 95% confidence intervals (blue bar) were shown. Sequences of ∼5.59 Mbp single-copy orthologous coding genes from each of 14 species were concatenated as an input sequence of MCMCtree. Four fossil calibration points (*A*–*D*) were obtained from a previous report (Perelman et al. 2011), and the nodal divergence dating (*E*) between primates and tree shrew was obtained from a previous study (Fan et al. 2013). The species figures were obtained from the Ensembl database (https://asia.ensembl.org/).

Based on the annotated NPM protein sequences, we constructed a highly resolved phylogenetic tree to estimate the evolutionary status of NPMs among thirteen primate species, including human being, four apes, five Old-World monkeys, three New-World monkeys, and tree shrew (an experimental animal close affinity to primate). The evolutionary tree suggested that NPM diverged from the southern PTM (*M. nemestrina*) ∼3.9 Ma, and split from RM (*M. mulatta*) ∼6.2 Ma (fig. 2). The phylogenetic characteristics of these primates are reflected in the diversity of evolutionary driving force, which may confer phenotypic and immunological differences between NPM and the other primates.

**Fig. 3.**
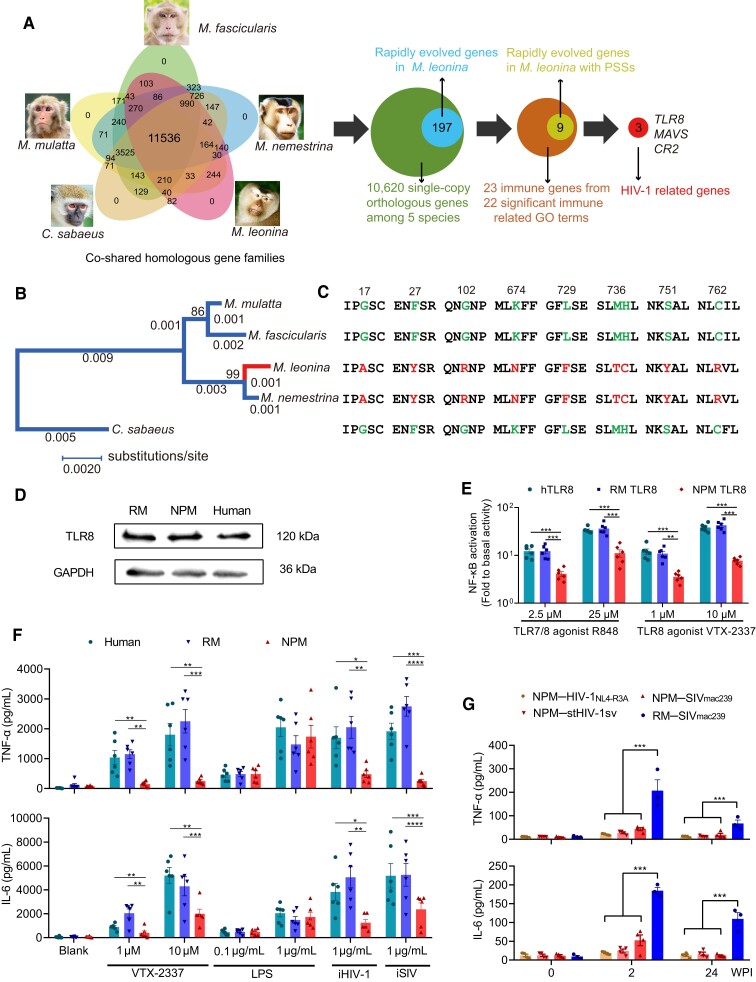
Genomics and functional analyses reveal that NPM-TLR8 is weaker than human (*h*)− or RM-TLR8 at inducing proinflammatory cytokines. (*A*) Bioinformatics pipeline for identifying rapidly evolving genes (PSGs) in NPM related to HIV-1 immunity. (*B*) Phylogenetic tree and (*C*) positively selected sites of TLR8 in the five most used SIV/HIV infection models among Old-World monkey species. (*D*) A representative photograph of IFI27 protein expressions in the PBMCs from NPMs, healthy RMs, and human donors. (*E*) Ability to activate NF-κB of NPM TLR8, compared with that of human and RMs TLR8 (one-way ANOVA). (*F*) Secretion of TNF-α and IL-6 from the blood of NPMs (*n* = 8) in response to the TLR8 agonist VTX-2337, TLR2/4 agonist LPS, heat inactivated HIV-1 (iHIV-1), and iSIV stimulation, compared with that from blood of healthy human donors (*n* = 8) or RMs (*n* = 8) (one-way ANOVA). (*G*) Plasma TNF-α and IL-6 levels in NPMs infected with HIV-1_NL4-R3*A*_ (*n* = 4), stHIV-1sv (*n* = 4), and SIV_mac239_ (*n* = 4), compared with that from RMs infected with SIV_mac239_ (*n* = 3) (one-way ANOVA). Data are presented as mean ± SEM (*P < 0.05, **P < 0.01, ***P < 0.001, ****P < 0.0001).

The continuous selective pressure from pathogens acts on host immune genes during primate evolution (Malfavon-Borja et al. 2013). Since the NPM is not a natural host of HIV-1 or SIV, we hypothesize that the history of some bacterial or viral diseases would have resulted in the rapid evolution of immune-related genes in the NPM, thereby shaping its present-day immune system to encounter specific pathogenic invasions, including HIV-1 and SIV. For instance, it is suspected that the *TRIM5α* and *cyclophilin* genes in PTMs had experienced the diversity selection from some unknown viruses, thus leading the generation of a new fusion gene *TRIM5-cyclophilin A*, which rendered the susceptibility of HIV-1 in PTMs (Newman et al. 2008; Virgen et al. 2008). To test this hypothesis, we compared the annotated NPM protein sequences with the other four closely related Old-World monkey species, including RMs (*M. mulatta*), cynomolgus macaque (*M. fascicularis*), southern PTMs (*M. nemestrina*), and African vervet monkey (*Chlorocebus sabaeus*). In total, we identified 11,536 homologous gene families and 10,620 single-copy one-to-one orthologous genes among the five Old-World monkey species (fig. 3*A*). To characterize the potential adaptive changes in functional genes, we utilized the branch-site model implemented in the likelihood ratio test to detect positively selected genes (PSGs) and identified 197 PSGs in the NPM (*M. leonine*) lineage (supplementary table S5, Supplementary Material online). Using gene ontology (GO) enrichment analyses, we found that the 197 PSGs could retrieve 29 genes, which were enriched in 22 immune-related GO functional categories, mainly concentrating on regulations of innate immune response, defense response to virus, interferon-alpha (IFNα) production, and interleukin-6 (IL-6) secretion (supplementary table S6, Supplementary Material online). This result exhibits evidence of pathogenic selection on innate immune-related genes in NPMs. In these 29 PSGs, we found that 9 genes (*TLR8*, *CR2*, *MAVS*, *CHRNA4*, *PGC*, *CLDN18*, *CLEC1B*, *ITGA4*, and *ENPP3*) have potential positively selected amino acid sites using Bayes Empirical Bayes analyses (supplementary table S7, Supplementary Material online). These nine genes may play a role in shaping the unique immune response against certain specific pathogens and, concomitantly or coincidentally, have an impact on HIV-1 susceptibility in NPMs. According to the literatures in HIV-1 research, the genes among them most likely related to HIV-1 infection are *TLR8*, *CR2*, and *MAVS* (fig. 3*A*). TLR8 is an endosomal pattern recognition receptor that senses single-stranded RNA (ssRNA) in viruses to initiate pro-inflammatory cytokine induction, maturation, and activation of T cells (Heil et al. 2004). *CR2* encodes complement C3d receptor 2, which is the main receptor for complement protein C3d, plays an essential role in adaptive immune response, and is associated with susceptibility to HIV-1 infection in humans (Meza et al. 2020). The mitochondrial antiviral protein mitochondrial antiviral signaling protein (MAVS) is a key player in the induction of antiviral responses. HIV-1 can block MAVS-dependent signaling via the mitotic kinase polo-like kinase 1 (Gringhuis et al. 2017). Mutations in MAVS cause it to be insensitive to HIV-1-dependent suppression and a low viral load during disease progression (Stunnenberg et al. 2020). Therefore, we assumed that these genes may play an important role in HIV-1 infection, and examined *TLR8* first to validate our hypothesis.

### Rapid Evolution of TLR8 Was Associated With Low Immune Activation in HIV-1 Infected NPMs

TLR8 can recognize ssRNA sequences originating from the HIV-1 long terminal repeat U5 region and concomitantly elicit proinflammatory signaling (Heil et al. 2004). TLR8 stimulation of host cells preferentially induces nuclear factor (NF)-κB-regulated cytokines, especially those associated with inflammation, such as TNF-α, IL-6, and IL-12, which contribute to HIV-induced immune activation (Gorden et al. 2005; Meås et al. 2020). The TLR8 variants with weak activity are related to slow decrease of CD4^+^ T cell and clinical progression of AIDS in HIV-1 infected individuals (Oh et al. 2008; Mackelprang et al. 2014). Based on the findings of genome sequencing, we confirmed the underlying difference in the TLR8 nucleotide sequences among NPMs, RMs, and humans using Sanger sequencing and identified 42 and 18 amino acid changes in TLR8 in NPMs compared with those in humans and RMs, respectively (supplementary fig. S3, Supplementary Material online), and in them the nine amino acid changes under positive selection were verified (fig. 3*B* and *C***)**. Using western blotting, we observed that the amino acid changes in TLR8 did not disturb its expression in the PBMCs from humans, RMs, or NPMs (fig. 3*D*). To validate the potential functional consequences, we overexpressed TLR8 genes from humans, RMs, and NPMs in HEK 293 T cells and treated them with TLR7/8 agonist R848 or TLR8 agonist VTX-2337 and found that both agonists induced dose-dependent NF-κB release. Upon the R848 and VTX-2337 stimulation, NPM-TLR8 invoked a significantly lower NF-κB activity than human- or RM-TLR8, implying that NPM-TLR8 is weaker than human- or RM-TLR8 at inducing inflammatory response (fig. 3*E*).

Then we checked the secretions of the representative NF-κB-regulated cytokines, TNF-α, and IL-6, in an in vitro coculture experiment. Because other nonviral TLRs, such as TLR2 and TLR4 can also elicit the inflammatory cytokine production, we cocultured whole bloods from healthy NPM, RM, and human donors with TLR8 agonist VTX-2337, and TLR2/4 agonist LPS and found that whole blood from NPMs induced lower TNF-α and IL-6 secretions than did RM or human blood following VTX-2337, but not LPS stimulation, suggesting that TLR8 is responsible for inducing a weaker inflammatory response in NPMs. Meanwhile, upon the inactivated stHIV-1sv or SIV_mac239_ stimulation, the whole bloods from NPMs also released a lower TNF-α and IL-6 than those from human and RM (fig. 3*F*). Correspondingly, the reduced plasma TNF-α and IL-6 concentrations were detected in the NPMs infected with HIV-1_NL4-R3A_, stHIV-1sv, and SIV_mac239_ (fig. 3*G*). Thus, HIV-1 and SIV evoked a dampened activity of TLR8 in NPMs both in vitro and in vivo. The weaker NPM-TLR8 function was supported by the lower activation of CD4^+^ T and CD8^+^ T cells following TLR8 agonist stimulation in vitro (supplementary fig. S4, Supplementary Material online) and HIV and SIV infection in vivo (fig. 1*D* and *E*; supplementary fig. S2, Supplementary Material online) and corroborated our previous findings that low immune activation and limited microbial translocation maintained superior intestinal integrity in SIV_mac239_-infected NPMs (Zhang et al. 2019). Collectively, these results provide a plausible explanation for the observed superior CD4^+^ T cell homeostasis and low immune activation in NPMs during HIV-1 and SIV infection.

### Downregulation of Inflammatory Response in HIV-1 Infected NPMs

**Fig. 4.**
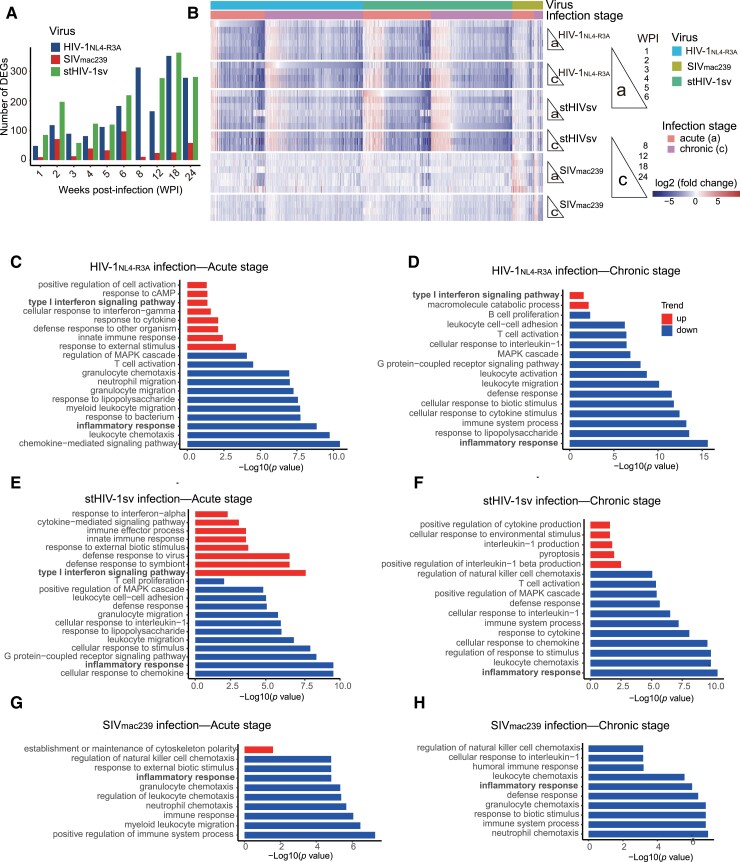
Transcriptome analyses of HIV-1 and SIV infection in NPMs. (*A*) The numbers of DEGs at each timepoint of infection compared with the preinfection (−2 wpi) in HIV-1_NL4-R3*A*_−, stHIV-1sv-, and SIV_mac239_-infected NPMs. (*B*) Heatmap showing the expressions of these DEGs following the three virus infections. The median values of the DEG expressions were shown. (*C–H*) Functional enrichment of up- and downregulated DEGs at acute and chronic stages. GO enrichments were adjusted for multiple comparisons (FDR < 0.05).

To gain further insight into the complex of molecular regulations in HIV-1 infection in NPMs, we conducted a comparative and longitudinal assessment of transcriptome changes in the PBMCs from HIV-1_NL4-R3A_−, stHIV-1sv-, and SIV_mac239_-infected NPMs through deep RNA sequencing at the time-points of −2, 1, 2, 3, 4, 5, 6, 8, 12, 18, and 24 wpi (fig. 1*A*). We first evaluated some differentially expressed gene (DEG) expressions by real time quantitative PCR (RT-qPCR), and validated that their expressions in the RNA sequencing were in line with those in the RT-qPCR assays (supplementary fig. S5, Supplementary Material online). Based on the large-scale RNA sequencing data, we observed highly concordant and extensive sharing of DEGs (fourfold change compared with pre-infection [−2 wpi]) in the two types of HIV-1 infections. Unexpectedly, most of these DEGs showed similar patterns of extensive downregulation during both acute and chronic infections. In stark contrast to the HIV-1 infections, the number of DEGs was much lower following SIV_mac239_ infection (fig. 4*A*). Among them, the upregulated DEGs were rarely shared with those in HIV infections, whereas the downregulated DEGs presented a similar pattern, but reduced to much less extents than those in HIV-1 infections (fig. 4*B*). Therefore, we first assessed the biological pathways of downregulated genes in all the three virus infections through GO analysis and found that they were enriched in the same term of “inflammatory response” at both acute and chronic infection stages (fig. 4*C*–*H*). The two HIV-1 strain infections shared 13 genes, including *CXCL2*, *IL10*, *CCR6*, *PDE2A*, *CCL3*, *CCR4*, *EPHA2*, *PTGES*, *FFAR2*, *IL8*, *IL6*, *CCL4*, and *IL1B* (supplementary table S8, Supplementary Material online). Among these genes, the expression of the inflammatory downstream mediators IL-10, IL-1B, IL-6, and IL-8 was significantly reduced, an interesting finding that was rarely observed in viral infection. This observation agreed with the slight changes of the plasma TNF-α and IL-6 concentrations in the HIV-1-infected NPMs (fig. 3*G*), the underlying mechanism for it remains largely unclear but may be partly attribute to or linked with the weaker function of NPM TLR8 (fig. 3*D*–*G*). Chemokine (C-C motif) ligand 3 (CCL3) plays a role in inflammatory responses by binding to receptors chemokine (C-C motif) receptor 1 (CCR1), CCR4, and CCR5 (Struyf et al. 2001), and their reduction may be associated with resistance to inflammation and HIV-1/SIV infection. Notably, in SIV_mac239_-infected NPMs, both the degree and extent of the downregulation of the inflammatory response were weaker than those in the two HIV-1 infections (fig. 4*C*–*H*; supplementary table S8, Supplementary Material online), which may be due to the more robust replication of SIV_mac239_ in NPMs.

Meanwhile, other downregulated GO terms, such as “T-cell activation,” “leukocyte chemotaxis,” and “leukocyte migration” were detected in HIV-1 infections at both acute and chronic infection stages (fig. 4*C*–*H*), implying that weak immune activation persisted. This result was consistent with the phenotype of T cells in the HIV-1-infected NPMs (fig. 1*D*–*E*; supplementary figs. S1 and S2, Supplementary Material online).

### Upregulation of Interferon Stimulated Genes After stHIV-1sv Infection

We next assessed the biological function of genes that were upregulated following HIV-1 infections. Gene enrichment analysis showed that the tendencies of HIV-1_NL4-R3A_ and stHIV-1sv was similar in that they induced an innate immune response to acute viral infection; in particular, they activated the type I interferon (IFN-I) signaling pathway, which is a key innate control against microbial invasion (Ivashkiv and Donlin 2014). Whereas SIV_mac239_ infection exhibited few genes that were significantly upregulated (fig. 4*C*–*H*), implying that IFN-I signaling may play a critical role in the inhibition of HIV-1 replication in the early HIV-1 infections in NPMs. In both HIV-1 strain infection, *IFI27*, *MX1*, *MX2* were enriched in the type I interferon signaling pathway (supplementary table S9, Supplementary Material online), they are all interferon-stimulated genes (ISGs).

**Fig. 5.**
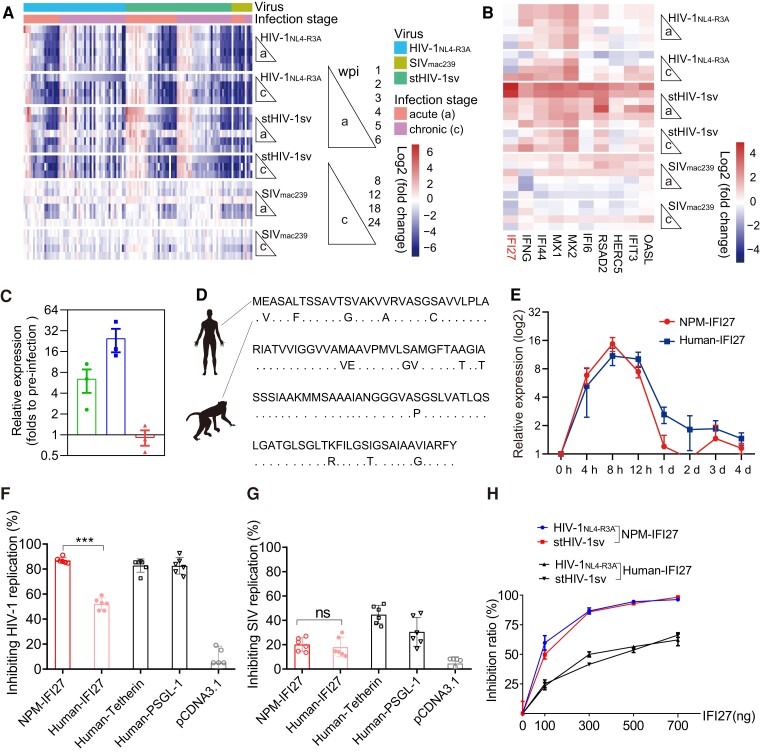
Regulation of ISGs following stHIV-1sv infection. (*A*) Heatmap showing the overlapping genes between ISGs and DEGs at each infection stage compared with the noninfection stage (−2 wpi) in PBMC samples infected by HIV-1_NL4-R3*A*_, stHIV-1sv, and SIV_mac239_. The median values of the DEG expressions were shown. (*B*) Overlap of ISGs and upregulated DEGs, 1 and 2 wpi, following stHIV-1sv infection. The heatmap showed 10 selected ISGs at each infection stage compared with the noninfection stage in samples infected with HIV-1_NL4-R3*A*_, stHIV-1sv, and SIV_mac239_. (*C*) Significant increases in IFI27 expression following HIV-1_NL4-R3*A*_ (*n* = 3), stHIV-1sv (*n* = 3), and SIV_mac239_ (*n* = 3) infection in NPMs at 2 wpi, validated by qRT-PCR. (*D*) Multiple amino-acid substitutions present in RM (*n* = 8) and NPM IFI27 (*n* = 8), compared with those in human IFI27 (*n* = 8). (*E*) IFI27 expression can be induced by IFN-α stimulation (in each group, *n* = 3). (*F*) Stronger anti-HIV-1 activity of NPM IFI27 compared with that of human IFI27, and (*G*) weaker anti-SIV activity of NPM and human IFI27*s* compared with that to HIV-1 (in each group, *n* = 6; one-way ANOVA, ns, no significant difference, ***P < 0.001). (*H*) NPM IFI27 showed a more robust dose-dependent inhibition of HIV-1 replication than human IFI27 (three repeats). In (*C*), and (*E–H*), data are presented as mean ± SEM.

Many of ISGs are identified as antiviral restriction factors and contribute to innate control of particular viruses (Ivashkiv and Donlin 2014). To further explore the role of other ISGs in HIV-1_NL4-R3A_ and stHIV-1sv infection, we screened the expression of 419 reported ISGs and identified the DEGs among them **(**fig. 5*A***)**. HIV-1_NL4-R3A_ and stHIV-1sv infections exhibited similar longitudinal patterns of ISG expression. Consistent with the tendency of DEGs (fig. 4*A*), most ISGs were downregulated even in acute infections (fig. 5*B*). However, some ISGs were upregulated following infection. Considering that stHIV-1sv is a more virulent strain than HIV-1_NL4-R3A_ in NPMs, and the ISGs are mostly upregulated at early time of infection in response to the viral invasion, we extracted the ISGs which were particularly upregulated at 1 wpi and 2 wpi during the stHIV-1 infection and retrieved a small cluster of them, including *IFI27, IFI6, MX1, MX2*, *RSAD2, IFI44, OASL, HERC5, IFNG*, and *IFIT3***(**fig. 5*B***)**. Among these genes, *MX2* is known as HIV-1 restriction factors (Kane et al. 2013); *RSAD2* (Nasr et al. 2012), *HERC5* (Paparisto et al. 2018), and *IFI44* (Power et al. 2015) have weak activities to inhibit HIV-1 replication; and *IFNG* is an anti-viral cytokine (Thobakgale et al. 2012). Their selective upregulations in stHIV-1sv infection, but not in SIV_mac239_ infection, could contribute to the observed suppression of stHIV-1sv replication in NPMs.

We then examined the anti-HIV-1 activity of the remaining genes, including *IFI27, IFI6, OASL,* and *IFIT3*, together with a gene *ARRDC4* as a negative control, and an HIV-1 restriction factor *Tetherin* as a positive control. Interestingly, when these genes were over-expressed in 293 T cells, NPM IFI27 showed the strongest anti-pHIV-1_NL4.3-R3A_ and pstHIV-1sv packaging activity, which was in a similar level with that of Tetherin, implying that IFI27 may play an important role in controlling HIV-1 replication in NPMs (supplementary fig. S6, Supplementary Material online).

### IFI27 in NPM Selectively Inhibited HIV-1 Replication

IFI27 is a mitochondrial localized protein belonging to the ISG12 gene family that is commonly induced by IFNs (Parker and Porter 2004) and participates in multiple biological processes, including apoptosis and congenital immunity (Labrada et al. 2002; Rosebeck and Leaman 2008). IFI27 has the capacity to inhibit hepatitis C virus replication (Chen et al. 2017), but whether it is related to HIV-1 replication remains unknown. In this study, IFI27 demonstrated the most considerable upregulations during acute stHIV-1sv infection (fig. 5*B*). Furthermore, IFI27 expression at 2 wpi in the PBMCs of infected NPMs was validated by qRT-PCR (fig. 5*C*). Genome sequences validated by Sanger sequencing showed that the protein sequences of IFI27 in NPM and RM were identical but showed multiple specific amino acid substitutions compared with those of humans (fig. 5*D*). Both human- and NPM IFI27 could be induced by exogenous IFN-α stimulation (fig. 5*E*), implying that it might be a host-specific ISG that participates in HIV-1 replication. To confirm this, we overexpressed human and NPM IFI27 in HOS CD4^+^ CCR5^+^ T cells and investigated their ability against HIV-1 and SIV replications. NPM IFI27 demonstrated selectively strong anti-HIV-1 but weak anti-SIV activities (fig. 5*F*–*FG*), supporting our finding of relatively low HIV-1 replication, while efficient SIV replication in NPMs (fig. 1*A*). Furthermore, NPM IFI27 demonstrated a much stronger inhibition against HIV-1 replication than human IFI27 (fig. 5*F*), as confirmed by a dose-dependent inhibition assay (fig. 5*H*). These results suggest that the anti-HIV-1 activity of IFI27 is host-specific.

**Fig. 6.**
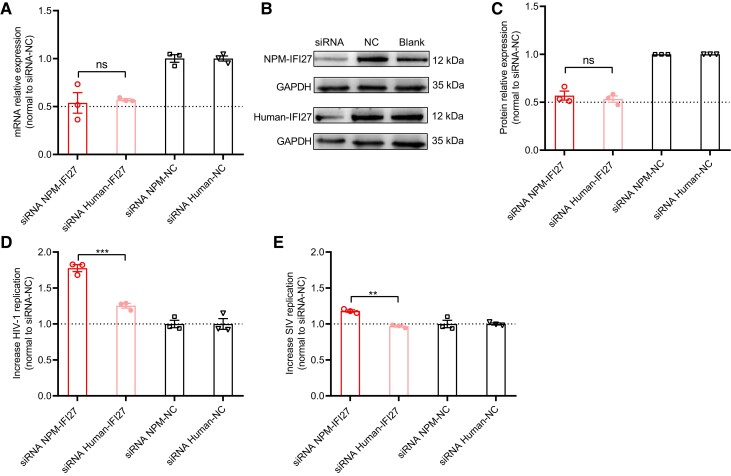
HIV-1 replication was increased by knocking down IFI27 in the PBMCs from healthy NPMs. Using siRNA, the levels of IFI27 expressions were knocked down in PBMCs from healthy NPM (*n* = 3) and human (*n* = 3) donors. After infection these cells with stHIV-1sv or SIV_mac239_, only stHIV-1sv replication increased in PBMCs from healthy NPMs, while there were little changes in stHIV-1sv replications in PBMCs from healthy NPMs and human donors. (*A*) The IFI27 mRNA relative expression levels. (*B*) A representative photograph of IFI27 protein expression detected by western-blotting. (*C*) Quantification of the bands in western-blotting. (*D*) The relative stHIV-1sv replication levels. (*E*) The relative stHIV-1sv replication levels. Data are presented as mean ± SEM (two-tailed paired *t*-test).

To further confirm it, using the siRNA targeting the IFI27, we observed that IFI27 mRNA expression was knocked down ∼50% in the PBMCs from healthy NPMs or humans (fig. 6*A*). Consequently, the IFI27 protein expressions were reduced 43% and 46% in the cells of NPMs and humans (fig. 6*B* and *C*). After applying the same amount of infective stHIV-1sv and SIV_mac239_ (MOI = 0.1) to these cells, we observed that stHIV-1sv replication significantly increased ∼78% in the cells from NPMs, but only ∼25% in human cells (fig. 6*D*). In turn, SIV_mac239_ replication was little changed in either cell types (fig. 6*E*). These results indicate that IFI27 is a host-specific HIV-1 restriction factor. Together, the significant upregulation of IFI27 and its selective anti-HIV-1 activity provide an explanation for the low HIV-1 replication in NPMs. It also suggests a potential target for genetic intervention in the NPMs for the adoption of a more appropriate HIV-1/AIDS model.

## Discussion

HIV-1 and its precursors have a long-term and complex coevolutionary history with different hosts. SIVs, precursors of HIV-1, have overcome numerous specific defensive barriers in their hosts to cross-species transmissions from monkeys to chimpanzees and eventually to humans. Phylogenetic analyses suggested that the viruses had experienced two stages of evolutionary events: 1) several different SIV strains, at least including SIV_gsn_, SIV_mon_, and SIV_mus_ in *Cerconpithecus* monkeys, had recombined to form a hybrid virus SIV_cpz_ in chimpanzees; 2) the SIV_cpz_ adapted to HIV-1 group M and transmitted in humans (Sauter and Kirchhoff 2019). In adaptations and cross-transmissions to new host species, the viral genes have evolved the ability to evade the defense mechanisms from the corresponding hosts, and in particular the viral accessory genes have obtained the antagonism to the host-specific restriction factors, facilitating the lowering of the species barriers. In turn, the high adaptability of the HIV-1/SIVs in each specific host has a negative fitness effect on a new host. As such, HIV-1 has evolved to be a highly human-specific retrovirus, hampering the development of a suitable nonhuman primate HIV-1/AIDS model. Nowadays, although RMs infected with SIV/SHIV have been widely used in HIV-1/AIDS research, these models have shown some shortcomings for anti-HIV-1 drug and vaccine applications.

Besides RMs, the PTMs have been used in HIV-1/AIDS research due to their HIV-1 susceptibility (Agy et al. 1992; Liao et al. 2007). Lacking of functional *TRIM5α* gene in PTMs has been well documented as a molecular mechanism underlying these observations, which lowers the barrier for HIV-1 cross-species infection in these monkeys (Liao et al. 2007; Newman et al. 2008; Virgen et al. 2008). To overcome another barrier from macaque restriction factor APOBEC3, an engineered HIV-1 derivative, stHIV-1sv, was constructed, and it presented a more robust replication in PTMs (Hatziioannou et al. 2009). However, neither HIV-1 (Agy et al. 1997) nor its derivative stHIV-1sv (Hatziioannou et al. 2009) are pathogenic in PTMs. Thereafter, the stHIV-1sv strain was adapted in vivo for several passages, and its descendants containing several mutations in structural and accessory genes such as *vpu*, *gag*-CA, or *env* have shown adaptations to other macaque-specific restriction factors tetherin, MX2, or the use of the viral coreceptor CCR5, respectively; they still cannot replicate well at chronic stage and induce AIDS in PTMs (Hatziioannou et al. 2014; Schmidt et al. 2019). Meanwhile, CD8^+^ T cells depletion in PTMs prior to HIV-1 infection can cause pathology but it is not a natural infection state (Hatziioannou et al. 2014; Schmidt et al. 2019). These results indicate that some unknown host-specific defense mechanisms still impede HIV-1 adaptation in this type of monkey. Therefore, revealing the unexplored barriers that inhibit HIV-1 fitness in PTMs will promote the development of HIV-1/AIDS animal model and extend the knowledge of HIV-1 evolution in primates.

The interactions between host and pathogen are major drivers of immune-related gene evolution, and species-specific genetic changes can help to understand the substitutions differentially impacting on the diverse outcomes of infectious diseases (Chapman and Hill 2012). Here, we observed that the NPM infected with HIV-1 showed a typical feature of low level of immune activation, we then employed large-scale comparative genome analysis to reveal potential genetic underpinning of HIV-1 infection in NPM. We revealed that the *TLR8* gene evolved to be functionally less efficient in NPM than in human and RM. As TLR8 has function to initiate pro-inflammatory cytokine induction, maturation and activation (Heil et al. 2004; Gorden et al. 2005; Meås et al. 2020), these changes on TLR8 may contribute to the observation that immunopathology is lacked in HIV-1-infected NPMs. This is similar to the natural SIV host species the sooty mangabey (*Cercocebus atys*), which does not develop AIDS following SIV infection (Chahroudi et al. 2012; Palesch et al. 2018). Specifically, functional genomics analysis in the Sooty mangabey has found that TLR4 special mutations could cause the attenuated production of pro-inflammatory cytokines, which is an important reason for the blunted immune activation in this monkey (Palesch et al. 2018). Considering that TLRs are an important class of pathogen-recognition receptors in hosts that recognized pathogen-associated molecular patterns in multiple exogenous viruses or bacteria (Wlasiuk and Nachman 2010), and they are subject to different evolutionary pressures from these pathogens, our study proposes another pattern of pathogen-recognition receptor in primates that may hinder HIV-1 or SIV pathogenicity. However, we should point out that in the present study, although TLR8 signaling was weaker in NPMs than in RMs or humans, it does not define a clear link between weak TLR8 signaling in NPMs and a lack of pathogenesis following HIV-1 or stHIVsv infection in this species and that further experiments directly evaluating this hypothesis would be required to make this connection.

Although NPM can be infected by HIV-1, viral replication is low, especially in chronic infection. To replicate well in a new host, HIV-1 must adapt and evade the known and unexplored species-specific antiviral factors. Understanding how the genetic variations and expressions that contribute to the suppression of HIV-1 adaptation in NPMs will provide us with prospects to develop this species as a suitable HIV-1/AIDS model and deepen our understanding of co-evolution between HIV-1 and the possible new hosts. In the present study, through longitudinal transcriptome analysis, we found that a number of ISGs were upregulated in early HIV-1 infections, and several of them have been demonstrated to have the capacities to impede HIV-1 replication. In particular, we identified *IFI27*, which exhibited more mutations in NPMs than in humans. This difference leads to an enhanced ability of NPM IFI27 to inhibit HIV-1 infection compared with human IFI27. Thus, we considered that NPM, as a nonnatural host of HIV-1 infection, could only have been made susceptible to HIV-1 in an evolutionary incident during which the *TRIM5α* gene was lost (Newman et al. 2008; Virgen et al. 2008). However, other intrinsic mechanisms of HIV-1 inhibition in ancestral macaques still remain, such as the IFN-I response and the subsequent multiple ISG productions, demonstrating a profound and combinatorial effect on the control of HIV-1 replication in NPM. Furthermore, the immune escape in HIV-1 evolution, well documented in humans, has not emerged in NPMs following several years of infection (Pang et al. 2017, 2018), which should be a short time for the adaptation of HIV-1 in this monkey. Thus, these factors act together to provide evidence as to why HIV-1 replicates at low levels and does not cause pathology in NPMs, and suggest that the barriers for successful adaptation and fitness of HIV-1 in NPMs are relatively high. However, the present study provides two potential hurdles to overcome for the future engineering or adaptation of HIV-1 derivatives in NPMs.

Taken together, our results provide a comprehensive resource for mechanistic and regulatory insight into HIV-1 infection in a new host, and these findings can be helpful in adapting NPM as a more appropriate HIV-1/AIDS model. Furthermore, the profile of molecular interactions between HIV-1 and NPMs will deepen the understanding of the innate and adaptive defense mechanisms from the host in the cross-species HIV-1 infection.

## Materials and Methods

### Animals Used in This Study

Twelve male NPMs and three male Chinese RMs were used in the present study. All subjects were aged 6–10 years and free from SIV, simian type-D retrovirus, and simian T-lymphotropic virus type 1 before HIV-1 or SIV infection. Animals were obtained from the Kunming Primate Research Centre, Kunming Institute of Zoology, Chinese Academy of Sciences and were housed and cared for in accordance with the American Association for Accreditation of Laboratory Animal Care standards. All experimental procedures were approved by the Ethics Committee of Kunming Institute of Zoology, Chinese Academy of Sciences (IACUC18008).

Two HIV-1 strains, HIV-1_NL4-R3A_ and stHIV-1sv, and SIV strain SIV_mac239_ were cultured and titrated as described previously (Pang et al. 2018). Four NPMs infected with HIV-1_NL4-R3A_ and four NPMs infected with stHIV-1sv were established as models of low level of replications and nonpathogenic infections. Four NPMs infected with SIV_mac239_ were established as a model of high level of replication and slow AIDS progression. Three RMs infected with SIV_mac239_ were established as a model of high level of replication and rapid AIDS progression. A tissue culture infectious dose 50% (TCID_50_) of 10^5^ HIV-1_NL4-R3A_, stHIV-1sv, or TCID_50_ of 5,000 SIV_mac239_ was inoculated into each macaque by intravenous injection.

Whole blood from each macaque was collected by venipuncture into an EDTA vacutainer tube. Plasma was collected from the whole blood at 1000 *g* for 20 min, and PBMCs were separated by Ficoll density gradient centrifugation and stored in liquid nitrogen for further analysis.

### Viral Load

The plasma viral loads from HIV-1_NL4-R3A_- and stHIV-1sv–infected NPMs were quantified using the COBAS TaqMan HIV-1 Test version 2.0 on the COBAS TaqMan 48 Analyzer (Roche Molecular Systems) with a detection limit of 20 copies/mL. The plasma viral loads from SIV_mac239_-infected NPMs or RMs were determined by real-time PCR (ABI ViiATM 7 Real-Time PCR System) as previously described (Zhang et al. 2016), with a detection limit of 50 copies/mL. Each sample in each viral infection was tested in three repeated wells, and the mean value of each sample was imported into Graphpad Prism 8.0.1. The data in each infection during 1–6 wpi or 7–69 wpi were compared with each other by two-way analysis of variance (ANOVA) test. The *P* values were calculated using the “Grouped analyses—Two-way ANOVA—Multiple comparisons—Compare column means (main column effect)” model.

### Flow Cytometry

Antibodies and reagents were purchased from BD Biosciences (Franklin Lakes, NJ, USA). The following anti-human flow cytometry antibodies cross-reactive with macaques were used: anti-CD3-phycoerythrin (PE)/-APC-Cy7 (clone SP34–2), anti-CD4-FITC/-PerCP-Cy5.5 (clone L200), anti-CD8-PE/Cy7 (clone RPA-T8), anti-CD20-PerCP-Cy5.5 (clone 2H7), and anti-HLA-DR-allophycocyanin (clone G46-6), anti-Ki67-PE (clone B56), and anti-CD38-FITC (clone AT-1). Absolute cell counts and antigen staining were performed using EDTA-anticoagulant whole blood. Sample preparation and lymphocyte immunophenotyping methods were described previously (Pang et al. 2018). Flow cytometry was performed on a BD FACSVerseflow cytometer (BD Biosciences), and data were analyzed using FLOWJO software (vX.0.7; TreeStar, Ashland, OR, USA).

### Genome Sequencing

Genomic DNA was extracted from blood samples of a male NPM (*M. leonina*) from Yunnan Province, China. The NPM genome was produced using an all-genome shotgun strategy on a next-generation sequencing platform. To reduce the risk of nonrandomness, 13 paired-end sequencing libraries with six insert sizes (250 bp, 450 bp, 2 kb, 5, 10, and 15 kb) were constructed and sequenced. In total, our sequencing data reached 425.38 Gbp with a high coverage of ∼141.79X.

### Genome Assembly and Annotation

ALLPATHS-LG (Gnerre et al. 2011) was used to produce a de novo NPM genome assembly. The high single-base accuracy of this assembly was evaluated based on the homozygous SNP ratio (6.99 e-04%). The completeness of our assembly was validated using different strategies, such as CEGMA (Core Eukaryotic Genes Mapping Approach) (http://korflab.ucdavis.edu/datasets/cegma/) and BUSCO (Benchmarking Universal Single-Copy Orthologs) (http://busco.ezlab.org/). For gene structure annotation, three pathways were applied and integrated. *De novo* predictions were inferred by integrating diverse software algorithms, including Augustus (http://bioinf.uni-greifswald.de/augustus/), GlimerHMM (http://ccb.jhu.edu/software/glimmerhmm/), SNAP (https://github.com/KorfLab/SNAP), Genscan (http://genes.mit.edu/GENSCAN.html), and Geneid (https://genome.crg.es/software/geneid/index.html). Homologous predictions were performed using eight downloaded genomes (*Homo sapiens*, *Mus musculus*, *Pan troglodytes*, *Pongo abelii*, *Gorilla gorilla*, *Rhinopithecus roxellana*, *M. mulatta*, and *M. nemestrina*) (http://asia.ensembl.org/). Transcriptomic data were also employed to analyze gene structure models using Cufflinks (http://cole-trapnell-lab.github.io/cufflinks/). EVidenceModeler (EVM) (https://github.com/EVidenceModeler/EVidenceModeler/) and PASA (https://github.com/PASApipeline/PASApipeline/) were utilized to obtain a nonredundant gene set based on three annotated pipelines.

### RNA Purification and RNA-sequencing

Total RNA from 5 × 10^6^ PBMCs from each infected macaque was extracted using TRIzol Reagent (Life Technologies, Carlsbad, CA, USA) and purified with RNeasy mini kits (Qiagen, Valencia, CA, USA) according to the manufacturers' protocols. RNA integrity was monitored on 1% agarose gel and assessed using the RNA Nano 6,000 assay kit of the Agilent Bioanalyzer 2,100 system (Agilent Technologies, Santa Clara, CA, USA). RNA purity was checked using a Nanophotometer spectrophotometer (IMPLEN, Los Angeles, CA, USA), and the concentration was measured using a Qubit RNA assay kit in a Qubit 2.0 Fluorometer (Life Technologies). For sequencing, 1.5 μg RNA per sample was used by an RNA Library Prep kit for Illumina (NEB, USA). Following cluster generation, library preparations were sequenced on an Illumina HiSeq 4,000 platform, and 150-bp paired-end reads were generated.

### Transcriptome Analyses

Read alignment and quality control: We obtained 99 transcriptomes spanning multiple infection stages from NPMs infected with different viruses. First, quality control was performed using fastp (Chen et al. 2018), any reads with N content exceeding 10% or low-quality (Q ≤ 5) bases exceeded 50% of the base total, and the corresponding paired reads were removed. Second, Tophat 2 (Kim et al. 2013) was used to align paired-end reads to the *M. leonina* reference genome, which was assembled in our lab. Lastly, further quality control was performed using Picard Tools (http://broadinstitute.github.io/picard/) with the command Collect RnaSeqMetrics. The sequencing metrics %mRNA Bases, % Intergenic Bases, and Median 5′ to 3′ Bias were selected to measure the sequence quality. To detect outliers, a quality z-score was calculated for each metric, and samples with low quality (z-score > 2 for % intergenic bases; z-score < −2 for %mRNA Bases, Median 5′ to 3′ Bias) were identified, and any sample with greater than one outlier value was removed due to sequencing quality concerns. Four samples were removed, and the remaining 95 samples were used for further analysis (supplementary table S10, Supplementary Material online).

Expression quantification: Cufflinks (Trapnell et al. 2010) was used to measure the expression of genes; cuffquant calculated the read count of each gene in each sample and generated an intermediate file that was used for further analyses with cuffnorm and cuffdiff; cuffnorm normalized the read count from cuffquant to FPKM (fragments per kilobase of exon model per million mapped reads). Gene expression levels were quantified using read count and FPKM.

Differential expression analyses: Cuffdiff (Trapnell et al. 2013) was used to perform pairwise differential expression analyses with default parameters between samples at each infection and noninfection stage. *P* values were corrected for multiple comparisons using the Benjamini–Hochberg procedure to estimate the false discovery rate (FDR). FDR < 0.05 and | log_2_ (fold change) | > 2 were required to define DEGs. We used g: Profiler (https://biit.cs.ut.ee/gprofiler/) (Reimand et al. 2016) to perform functional enrichment analyses of DEGs, and *P*-values were corrected for multiple comparisons using the Benjamini–Hochberg procedure to estimate the FDR.

ISGs were selected at (https://www.ncbi.nlm.nih.gov/gene) with “(Interferon stimulated genes) AND “Homo sapiens“[porgn: txid9606]”. We included 419 ISGs of human origin in the study.

### Antiviral Activity of IFI27

To screen the anti-HIV-1 activities of ISGs, the CDS regions of *ARRDC4*, *MX1*, *OSAL*, *IFIT3, IFI27*, and *IFI6* from NPMs, and *IFI27*, *IFI6, Tetherin,* and *PSGL-1* from humans were synthesized into the *Bam*H1 and *Eco*RI restriction sites of the pcDNA3.1(+) vector (Invitrogen) and then sequenced (Tsingke, Shanghai, China) for verification. To evaluate the anti-HIV-1 activities of these ISGs, HEK293T cells were co-transfected with 200 ng of each expression vector and 200 ng of pHIV-1_NL4-R3A_ or pHIV-1_NL4-R3A_ in 24-well plates using jetPRIME transfection reagent. *Tetherin* and the pcDNA3.1 vectors were used as positive and negative controls, respectively. 48 h post-transfection, the HIV-1 p24 protein concentration in the culture supernatant was determined by an ELISA assay (SinoBiological, Beijing, China).

To verify whether IFI27 can be induced by IFN-α, PBMCs (1 × 10^6^/well in 24-well plates) from three healthy human or NPM donors were co-cultured with 200 IU IFNα (Sigma). Cells were collected at different time points and total RNA was extracted using TRIzol reagent (Life Technologies), and cDNA was generated with a PrimeScript RT reagent Kit with gDNA Eraser (Takara). Expression levels of IFI27 were determined by the real-time qPCR assays described above.

After the anti-HIV-1 packaging activities of IFI27 were screened out, HOS CD4^+^ CCR5^+^ cells were transfected with 200 ng of IFI27 or IFI6-expressing vector from NPMs or humans (pcDNA3.1-NPM-IFI27, pcDNA3.1-human-IFI27) in 24-well plates using jetPRIME transfection reagent. Tetherin and PSGL-1 from humans cloned into the pcDNA3.1 vector were used as positive controls, and the pcDNA3.1 vector was used as a negative control. 24 h post-transfection, the cells were infected with HIV-1_NL4-R3A_ or stHIV-1sv strains at a multiplicity of infection (MOI) of 0.1 (infectious titers were determined based on TZM cells). At 6 h post-infection, the cells were washed three times with PBS at 1000 g/8 min to remove the uninfected viruses, and a new culture medium was added. After 48 h of infection, the HIV-1 particles in the culture supernatant were determined using the real-time qPCR assay described above, and the inhibition of viral replication by each gene of interest was calculated.

To determine the species-specific anti-HIV-1 activity of IFI27, we overexpressed NPM IFI27 and human IFI27 in HOS CD4^+^ CCR5^+^ cells, and determined the reduction in viral replication. Briefly, a series of pcDNA3.1-NPM IFI27 or pcDNA3.1-human IFI27 (0 ng, 100 ng, 300, 500, and 700 ng) was transfected into HOS CD4^+^ CCR5^+^ cells in 24-well plates. At 24 h post-transfection, the cells were infected with stHIV-1sv or SIV_mac239_ strains at an MOI of 0.1 and washed at 6 h later. At 48 h post-infection, the different antiviral activities of human IFI27 and NPM IFI27 were evaluated as described above.

In contrary, we knocked down NPM IFI27 and human IFI27 in PBMCs from NPMs and humans, and examined the increase in viral replication. Briefly, PBMCs (2 × 10^6^/mL) from 3 healthy NPMs and human donors were cultured in RPMI 1,640 medium with 10% FBS in 6-well plates, after stimulated with ConA (5 μg/mL) and IL-2 (50 IU/mL) for 2 days, the cells were transfected with siRNA targeting NPM IFI27, human IFI27 and siRNA-NC. After 48 h, the transfected cells from one plate were collected for real-time qPCR and Western blot analyses. The sequence of the siRNA targeting NPM IFI27 is followed by the sense: 5′-GGAUUGCUACAGUCGUGAUTT-3′, antisense: 5′-AUCACGACUGUAGCAAUCCTT-3′. The sequence of the siRNA targeting human *IFI27* is followed by the sense: 5′-CCUCUGGCUCUGCCGUAGUUU-3′, antisense: 5′− AAACUACGGCAGAGCCAGAGG-3′. Total RNA extraction, real-time qPCR and Western blot were evaluated as described above. Primers for amplifying NPM *IFI27* or human *IFI27* were designed based on each mRNA sequence. NPM *IFI27* was normalized to that of the *RPL13A* mRNA, and human *IFI27* was normalized to that of the *GAPDH* mRNA (supplementary table S11, Supplementary Material online).

In the other plate, 48 h after siRNA transfection, the PBMCs were infected with stHIV-1sv or SIV_mac239_ strains at a MOI of 0.1. The cells were washed 6 h later, and the different increases of viral replications from human IFI27 or NPM IFI27 knocking down were evaluated as described above.

Additional details are described in the Supplementary Materials and Methods, Supplementary Material online.

## Supplementary Material

msad101_Supplementary_Data

## Data Availability

The northern pig-tailed macaque (NPM, *Macaca leonina*) genome assembly and annotation data have been deposited at the open scientific database, figshare (https://figshare.com/) with a Digital Object Identifier (DOI: https://doi.org/10.6084/m9.figshare.21694727.v1). The longitudinal transcriptome data in PBMCs from HIV-1_NL4-R3A_−, stHIV-1sv-, and SIV_mac239_-infected NPMs are available at NCBI BioProject (accession number: PRJNA757211). All the other experimental data are included in the main text or the Supplementary Materials, Supplementary Material online. ***Conflict of interest statement.*** Authors declare that they have no competing interests.
