# Supplementary material for: Genomic Evidence for the Nonpathogenic State in HIV-1–Infected Northern Pig-Tailed Macaques
Source: Mol Biol Evol. 2023 May 3;40(5):msad101. doi: 10.1093/molbev/msad101 (PMC10182734; doi:10.1093/molbev/msad101)
Supplement: msad101_Supplementary_Data [file msad101_supplementary_data.docx]

Supplementary information for

**Genomic evidence for nonpathogenic state in HIV-1-infected northern pig-tailed macaques**

Wei Pang, Yong Shao, Xiao-Lin Zhuang, Ying Lu, Wen-Qiang He, Hong-Yi Zheng, Rong Xin, Ming-Xu Zhang, Xiao-Liang Zhang, Jia-Hao Song, Ren-Rong Tian, Fan Shen, Yi-Hui Li, Zu-Jiang Zhao, Dong-Dong Wu* & Yong-Tang Zheng*

Correspondence to: wudongdong@mail.kiz.ac.cn; [zhengyt@mail.kiz.ac.cn](mailto:zhengyt@mail.kiz.ac.cn)

**This file includes:**

Supplementary Materials and Methods

Figures S1 to S6

Tables S1 to S11

**Supplementary Materials and Methods**

***Evolutionary analyses***

Twelve primate genomes and an outgroup species genome were downloaded from NCBI Assembly Database (https://www.ncbi.nlm.nih.gov/assembly/) including human being (*Homo sapiens*), chimpanzee (*Pan troglodytes*), western gorilla (*Gorilla gorilla*), sumatran orangutan (*Pongo abeliii*), and northern white-cheeked gibbon (*Nomascus leucogenys*), rhesus monkey (*Macaca mulatta*), cynomolgus macaque (*Macaca fascicularis*), southern pig-tailed macaque (*M. nemestrina*), African vervet monkey (*Chlorocebus sabaeu*s), white-tufted-ear marmoset (*Callithrix jacchus*), small-eared galago (*Otolemur garnettii*), gray mouse lemur (*Microcebus murinus*), and Chinese tree shrew (*Tupaia belangeri*). The single-copy one-to-one orthologous genes among 14 species were identified by INPARANOID (Ostlund et al. 2010) and MULTIPARANOID (Alexeyenko et al. 2006) algorithms using default parameters. These single-copy orthologous genes were aligned using PRANK (Löytynoja 2014) with parameters (-d=$file -o=$file.fas -f=fasta -F -codon -noxml -notree -nopost). After the sequence quality trimming based on the GBLOCKS (Castresana 2000) algorithm (Parameter setting: /Gblocks $i -t=c -b4=5), these single-copy sequences from 1 and 2 positions of codons across 14 species were further concatenated as an input sequence to produce a highly reliable species tree using RAxML (Stamatakis 2014) with 100 bootstrap replicates under the GTRGAMMA model. Meanwhile, they also were performed to generate a divergence time evaluation based on the MCMCtree algorithm in PAML (Yang 2007). Our fossil calibration points were obtained from the previous studies (Perelman et al. 2011; Fan et al. 2013). The single-copy orthologous genes across five species (*M. mulatta*, *M. fascicularis*, *M. leonina*, *M. nemestrina*, and *Chlorocebus sabaeus*) were extracted, and coding sequences for each gene were aligned using PRANK (Löytynoja 2014). The GBLOCKS (Castresana 2000) algorithm further identified conserved blocks and removed low-quality aligned regions in a multiple sequence alignment for each gene. Based on a five-species guided tree topology, the codeml algorithm under a free-ratio model in PAML (Yang 2007) was applied to calculate the parameters (i.e., N, S, *d*_N_/*d*_S_, *d*_N_, *d*_S_, N**d*_N_, and S**d*_S_) for each gene along with diverse branches. Furthermore, the d_N_/d_S_ of each Gene Ontology (GO) related to biological processes was integrated by the *d*_N_/*d*_S_ of GO-related genes. The significance of positively selected genes was calculated using the χ^2^ test, with *p*<0.05 indicating significance. The Bayes empirical Bayes algorithm in PAML (Yang 2007) was applied to infer the posterior probabilities for potential positively selected sites.

The evolutionary tree of TLR8 was inferred using the maximum likelihood method and the Tamura-Nei model (Tamura and Nei 1993). The tree with the highest log-likelihood was shown. The percentage of trees with 200 bootstraps in which the associated taxa clustered together was shown next to the branches. Initial trees for the heuristic search were automatically obtained by applying Neighbor-Join and BioNJ algorithms to a matrix of pairwise distances estimated using the Tamura-Nei model, and then selecting the topology with the best log-likelihood value. This analysis involved five nucleotide sequences. The codon positions included were 1st, 2nd, and 3rd. There were 1617 positions in the final dataset. Evolutionary analyses were conducted using MEGA X (Kumar et al. 2018).

***Re-sequencing of TLR8, IFI27, and IFI6 among NPMs, RMs, and humans***

To verify the coding sequence (CDS) differences in *TLR8*, *IFI27*, and *IFI6* among NPMs, RMs, and humans, PBMCs (2×10^6^) from eight individuals of each species were extracted using TRIzol reagent (Life Technologies) and reverse transcribed into cDNA using the PrimeScript RT reagent kit with gDNA Eraser (Takara, Beijing, China). Primers were designed to flank three CDS areas of three genes of interest: *TLR8*-F: 5′-CTGCAAGTTACGGAATGAAAAATTAG-3′, *TLR8*-R: 5′-GTCATTCCTTTGCATCTTTATTATG-3′, *IFI27*-F: 5′-ACATTCTCAGGAACTCTCCTTCT-3′, *IFI27*-R: 5′-AGGTATATTTGGGATAGTTGGCT-3′, *IFI6*-F: 5′-CTGTCTAATAAGTCTAGCGACGG-3′, *IFI6*-R: 5′-TGTTGGGGACAGTGATAGAGAA-3′. Ex Taq DNA Polymerase (Takara) was used to amplify PCR products under the following conditions: 95 °C for 1 min; 95 °C for 30 s, 60 °C for 2 min (*TLR8*) or 60 °C for 30 s (*IFI27* and *IFI6*), 72 °C for 30 s; 72 °C for 8 min. PCR products were cloned into the pMD19-T simple vector (TaKaRa) and subjected to Sanger sequencing (Majorbio, Shanghai, China).

***Western blot analyses for TLR8 and IFI27***

The PBMCs (2×10^6^) from NPMs and Human donors were washed in PBS and the proteins were extracted using membrane and cytosol protein extraction kit (Beyotime, Shanghai, China) and quantified using the BCA kit (Beyotime). The protein sample (50 μg each) was loaded for electrophoresis and membrane transferring. After incubated with the rabbit polyclonal antibody to TLR8 or IFI27 (Affinity Biosciences, Wuhan, China) at 4 °C overnight, the membrane was incubated with the secondary antibody containing HRP labeled GAPDH (Abclonal, Wuhan, China) and followed by coloration.

***NF-κB luciferase reporter assay***

The CDS regions of TLR8 from human, NPM and RM were cloned into the *Bam*H1and *Eco*RI restriction sites of the pcDNA3.1 (+) vector (Invitrogen) and then sequenced (Tsingke, Shanghai, China) for verification. HEK293T cells were co-transfected with 200 ng of pcDNA3.1 vector expressing the indicated TLR8 allele, 200 ng of a firefly luciferase reporter construct under the control of three NF-κB binding sites (pGL4.32), and 50 ng of a Gaussia luciferase construct (pRL-TK) for normalization in 24-well plates using jetPRIME transfection reagent according to the manufacturer’s protocols (polyplus-transfection, Illkirch, France). In addition, 200 ng of a constitutively active mutant of IKKβ expression vector as an inducer of NF-κB and 200 ng of a pcDNA3.1 (+) vector were used to substitute TLR8 expression vector as positive and negative controls, respectively. Twenty-four hours post transfection, different concentrations of TLR7/8 agonist R848 (InVivoGen, San Diego, CA, USA) and TLR8 agonist VTX2337 (TargetMol, Boston, MA, USA) were added to the wells, except for IKKβ. 48 h post-transfection, luciferase activity was determined using the dual-luciferase reporter assay kit (Promega, Madison, USA).

**In vitro *whole blood stimulation and cytokine measurement***

Eight blood samples from healthy human donors were collected from our laboratory. Informed consent was obtained from each donor. Eight blood samples from healthy NPMs or RMs were obtained from the Kunming Primate Research Centre. Whole blood was collected in EDTA vacutainers, and diluted 1:4 with RPMI 1640 medium within 6 h (Palesch et al. 2018). Then, 180-μL aliquots were added to 96-well, flat-bottom microtiter plates. For TLR ligand stimulation, 20 μL of the diluted TLR7 agonist R837, TLR7/8 agonist R848 (Invivogen), TLR8 agonist VTX2337 (TargetMol) and TLR2/4 agonist LPS (*Escherichia coli* 055: B5, Sigma-Aldrich, Saint Louis, MI, USA) dissolved in RPMI 1640 medium were added into each well. For heat-inactivated stHIV-1 and SIV_mac239_ stimulation, 1 μg/mL (p24 or p27 protein concentration) stHIV-1 or SIV_mac239_ particles were inactivated at 56 °C for 30 min, and 20 μL of each aliquot was added. The suspensions were mixed and incubated at 37 °C.

After co-culture for 16 h, the mixture in each well was collected separately and centrifuged at 400 *g* for 10 min, and 150 μL of supernatant was removed and stored at −20 °C for cytokine measurement. The TNF-α and IL-6 concentrations in the supernatant from whole blood stimulation or the plasma from infected NPMs and RMs were determined using the Rhesus Macaque TNF-alpha Quantikine ELISA Kit (R&D Systems, Minneapolis, MN, USA) and Monkey Interleukin 6 ELISA Kit (Cusabio, Wuhan, China).

In another parallel experiment, after 3 days of co-culture, the cells from each well were collected separately and centrifuged at 400 *g* for 10 min for Flow cytometry analyses.

***Real-time qPCR assays for verification of transcriptional analysis***

PBMCs (2×10^6^) at 2 weeks pre-infection and 2 weeks post-infection were collected from HIV-1_NL4-R3A_-, stHIV-1sv-, or SIV_mac239_-infected NPMs, and total cellular RNA was extracted using TRIzol reagent (Life technologies). cDNA was generated using the PrimeScript RT reagent kit with gDNA Eraser (Takara). Real-time qPCR reactions were performed on a ViiA7 Real-Time PCR System using SYBR Premix Ex Taq II. The primers for amplifying the ISGs were designed according to the NPM mRNA sequences (supplementary tables S11, Supplementary Material online). Expression levels of the genes of interest were analyzed using the comparative cycle threshold (Ct) method, where Ct is the cycle threshold number normalized to that of the *RPL13A* mRNA. Fold-change was calculated using the 2^−ΔΔCt^ method by dividing the normalized quantity of post-infection samples by that of the preinfection samples.

**Reference:**

Alexeyenko A, Tamas I, Liu G, Sonnhammer EL. 2006. Automatic clustering of orthologs and inparalogs shared by multiple proteomes. *Bioinformatics.* 22(14): e9-15.

Castresana J. 2000. Selection of conserved blocks from multiple alignments for their use in phylogenetic analysis. *Mol Biol Evol.* 17(4): 540-552.

Fan Y, Huang ZY, Cao CC, Chen CS, Chen YX, Fan DD, He J, Hou HL, Hu L, Hu XT, et al. 2013. Genome of the Chinese tree shrew. *Nat Commun.* 4: 1426.

Kumar S, Stecher G, Li M, Knyaz C, Tamura K. 2018. MEGA X: Molecular Evolutionary Genetics Analysis across Computing Platforms. *Mol Biol Evol.* 35(6): 1547-1549.

Löytynoja A. 2014. Phylogeny-aware alignment with PRANK. *Methods Mol Biol.* 1079: 155-170.

Ostlund G, Schmitt T, Forslund K, Köstler T, Messina DN, Roopra S, Frings O, Sonnhammer EL. 2010. InParanoid 7: new algorithms and tools for eukaryotic orthology analysis. *Nucleic Acids Res.* 38(Database issue): D196-203.

Palesch D, Bosinger SE, Tharp GK, Vanderford TH, Paiardini M, Chahroudi A, Johnson ZP, Kirchhoff F, Hahn BH, Norgren RB, et al. 2018. Sooty mangabey genome sequence provides insight into AIDS resistance in a natural SIV host. *Nature.* 553(7686): 77-81.

Perelman P, Johnson WE, Roos C, Seuánez HN, Horvath JE, Moreira MA, Kessing B, Pontius J, Roelke M, Rumpler Y, et al. 2011. A molecular phylogeny of living primates. *PLoS Genet.* 7(3): e1001342.

Stamatakis A. 2014. RAxML version 8: a tool for phylogenetic analysis and post-analysis of large phylogenies. *Bioinformatics.* 30(9): 1312-1313.

Tamura K, Nei M. 1993. Estimation of the number of nucleotide substitutions in the control region of mitochondrial DNA in humans and chimpanzees. *Mol Biol Evol.* 10(3): 512-526.

Yang Z. 2007. PAML 4: phylogenetic analysis by maximum likelihood. *Mol Biol Evol.* 24(8): 1586-1591.


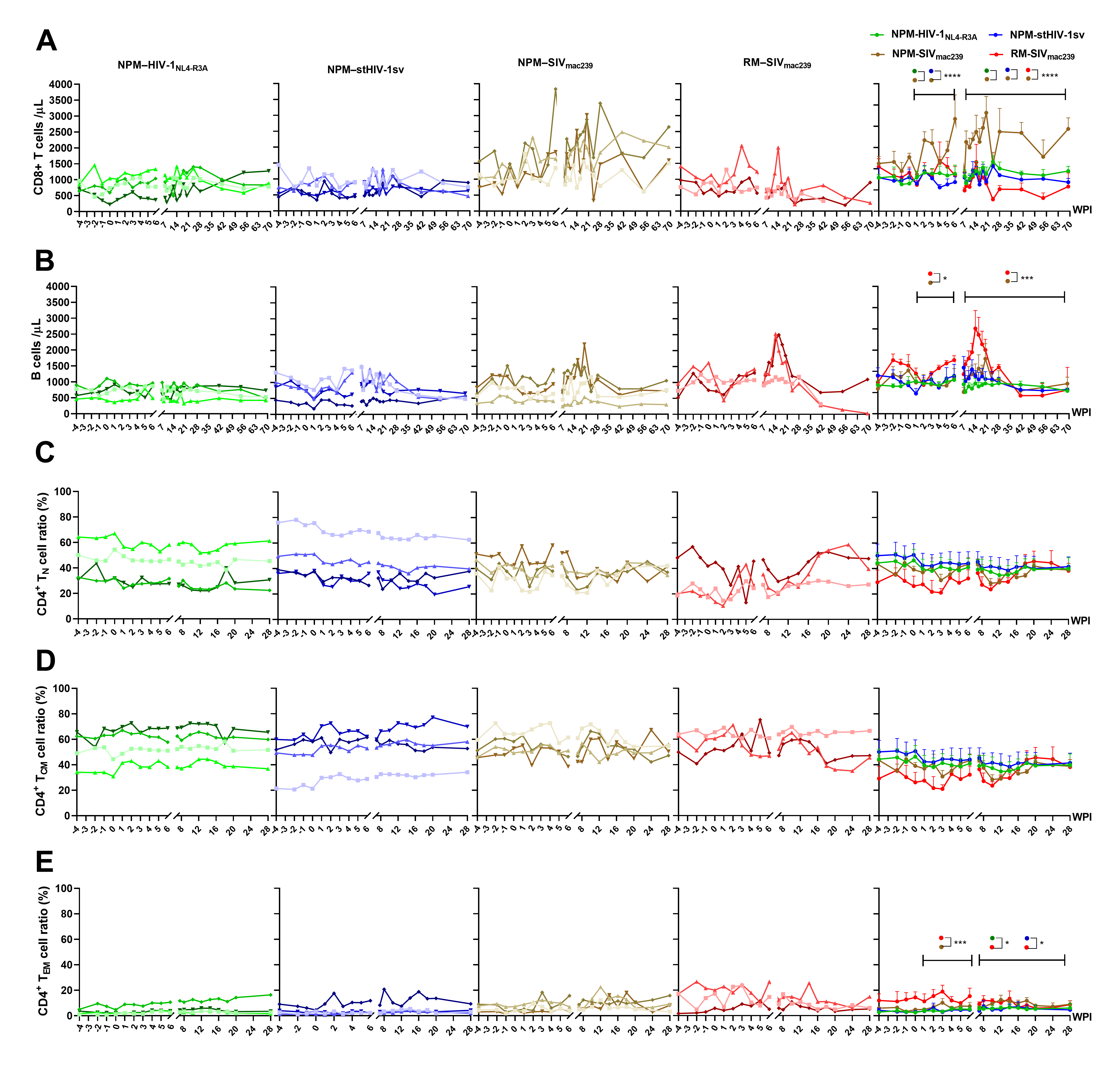


**Fig. S1. The kinetic changes of main subset of immune cells in HIV and SIV infected northern pig-tailed macaques (NPMs) or rhesus macaques (RMs).** The counts of CD8^+^ T cells (**A**) and B cells (**B**), and the ratio of CD4^+^ T naïve (CD4^+^ T_N_) (**C**), CD4^+^ T central memory (CD4^+^ T_CM_) (**D**) and CD4^+^ T central memory (CD4^+^ T_EM_) (**E**) in total CD4^+^ T cells. WPI: weeks post infection. Each shape in different infections represents each macaque, and in the last panel of **B**–**E**, data are presented as mean with SEM, and the data in each infection during 1-6 wpi or 7-69/7-28 wpi were compared to each other by a two-way ANOVA test (* *p*<0.05, ** *p*<0.01, *** *p*<0.001, **** *p*<0.0001), the same was followed in figure S2.


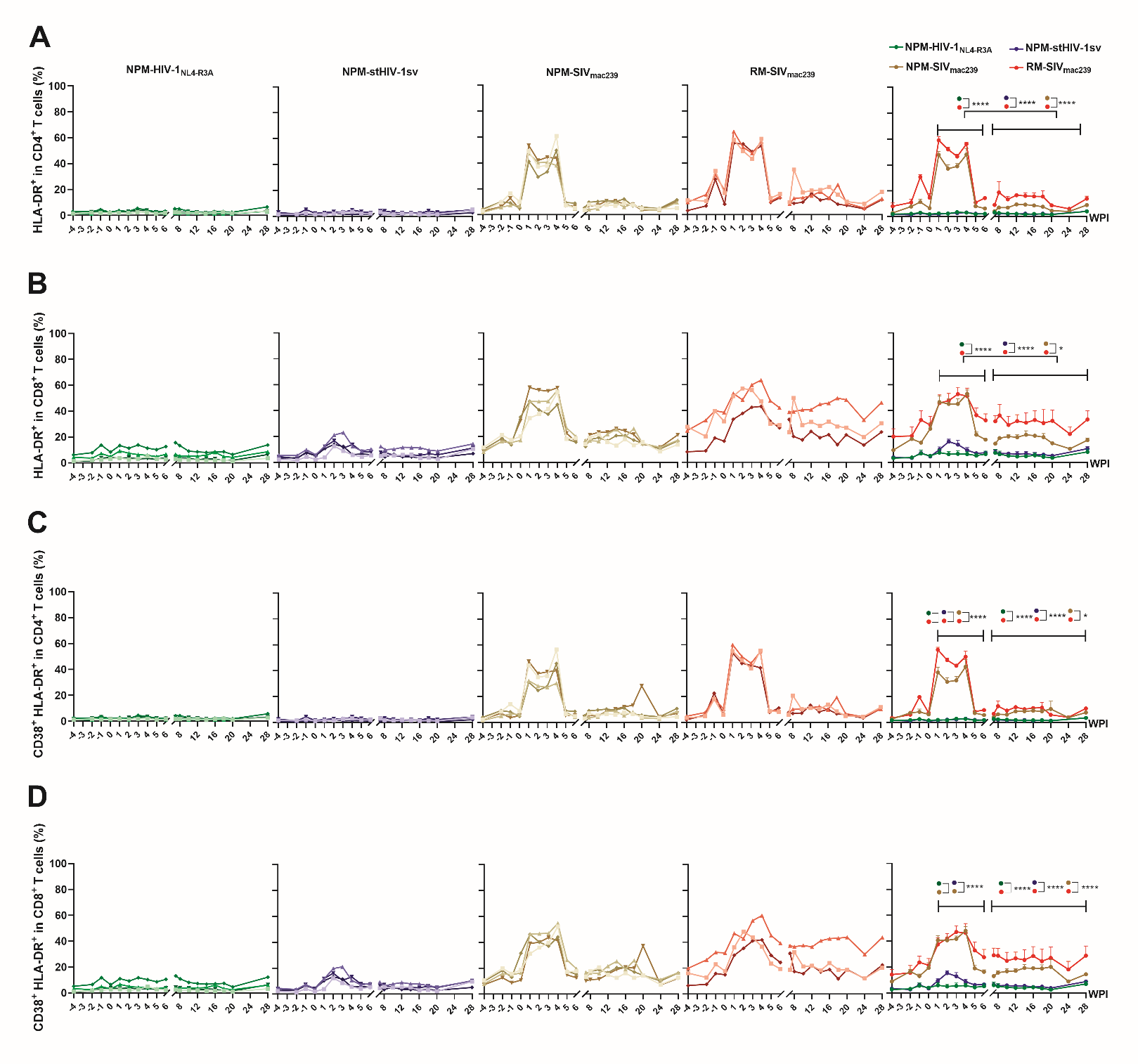


**Fig. S2. T cell activation in peripheral blood following HIV-1 or SIV infection in northern pig-tailed macaques (NPMs) or rhesus macaques (RMs).** The activation of CD4^+^ T cells (**A**, **C**) and CD8^+^ T cells (**B**, **D**) in peripheral blood that express HLA-DR (**A**, **B**) or both HLA-DR and CD38 (**C**, **D**) after HIV and SIV infection in NPMs or SIV infection in RMs. Each shape in different infections represents each macaque (two-way ANOVA test between each group).

**
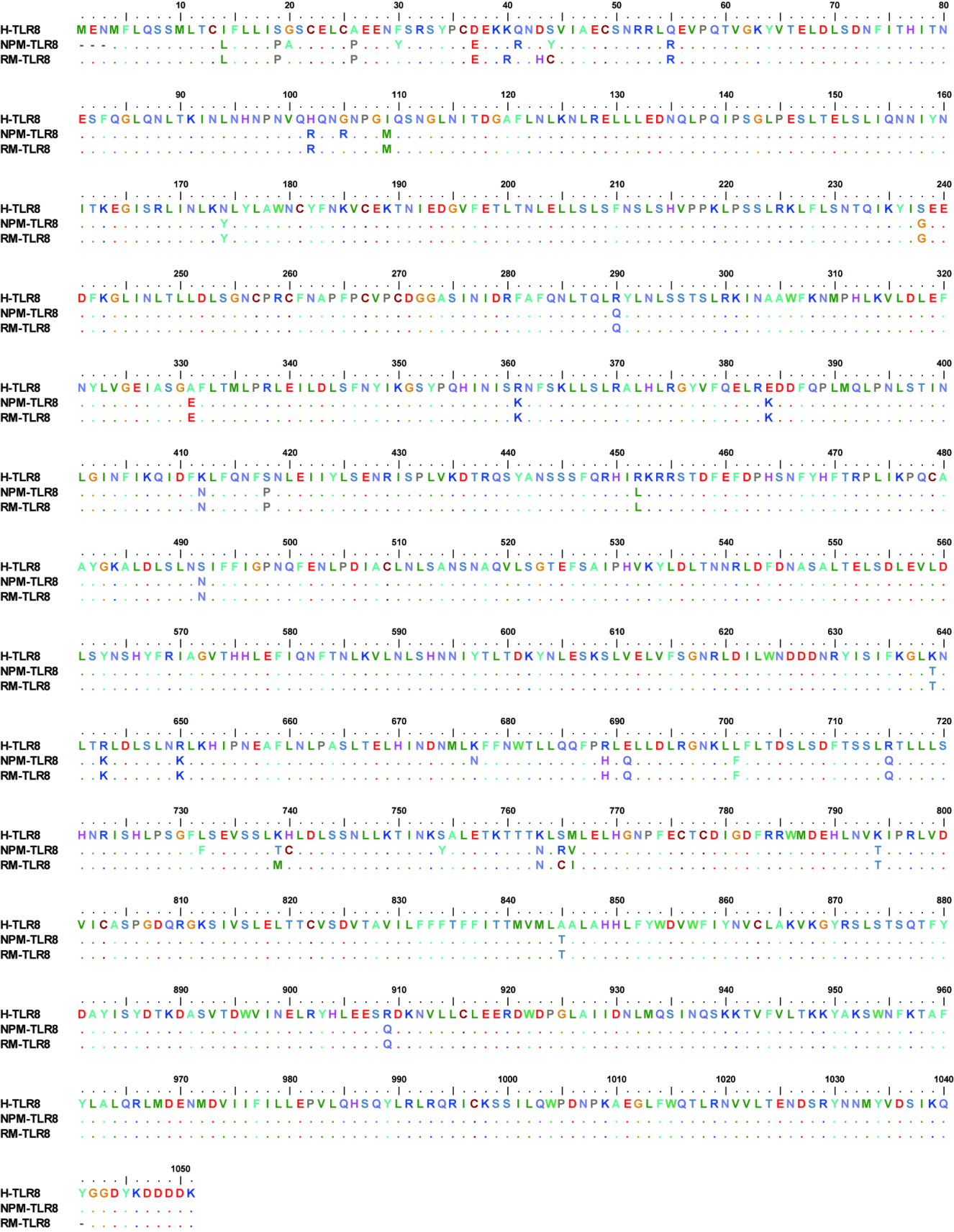
**

**Fig. S3. More specific amino-acid substitutions were present in northern pig-tailed macaque- (NPM-) TLR8, than human- (H-) and rhesus macaque- (RM-) TLR8.** In each species, the CDS regions of TLR8s from 8 individuals were sequenced and they were identical.

**
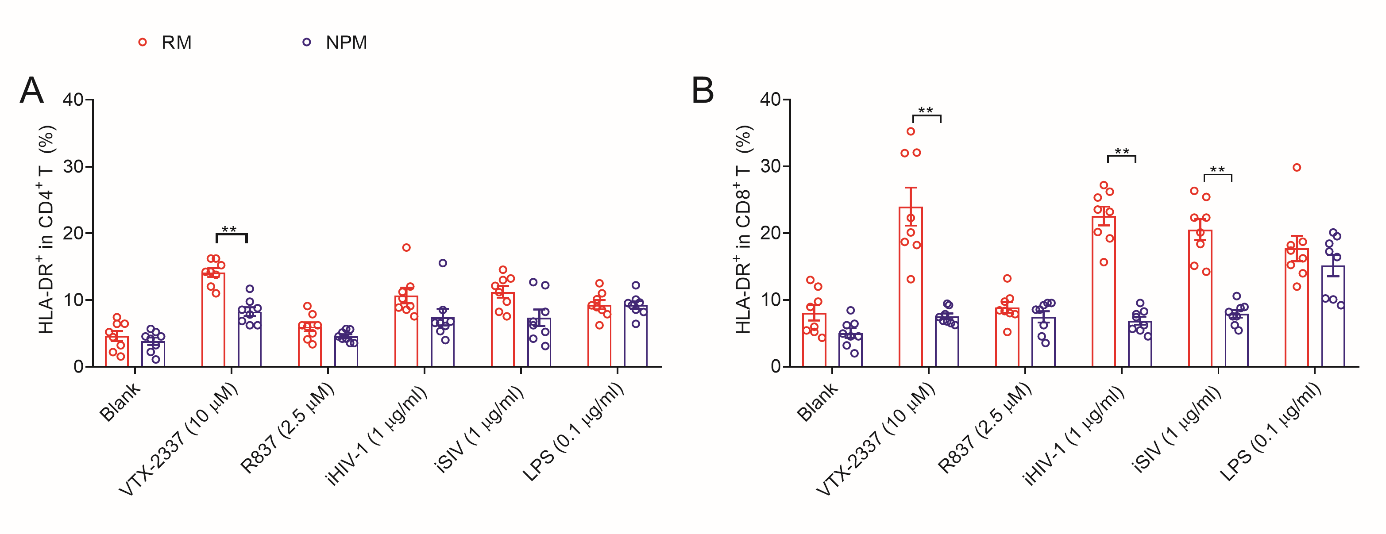
**

**Figure S4. T cell activation in peripheral blood from healthy northern pig-tailed macaques (NPMs) or rhesus macaques (RMs) upon various TLR agonists stimulation.** The activation of CD4^+^ T cells (**A**) and CD8^+^ T cells (**B**) in peripheral blood that express HLA-DR (Wilcoxon test). VTX-2337 is a TLR8 agonist, R837 is a TLR7 agonist, LPS is a TLR2/4 agonist, iHIV-1 means HIV-1 heat inactivated, and iSIV means heat inactivated iSIV.


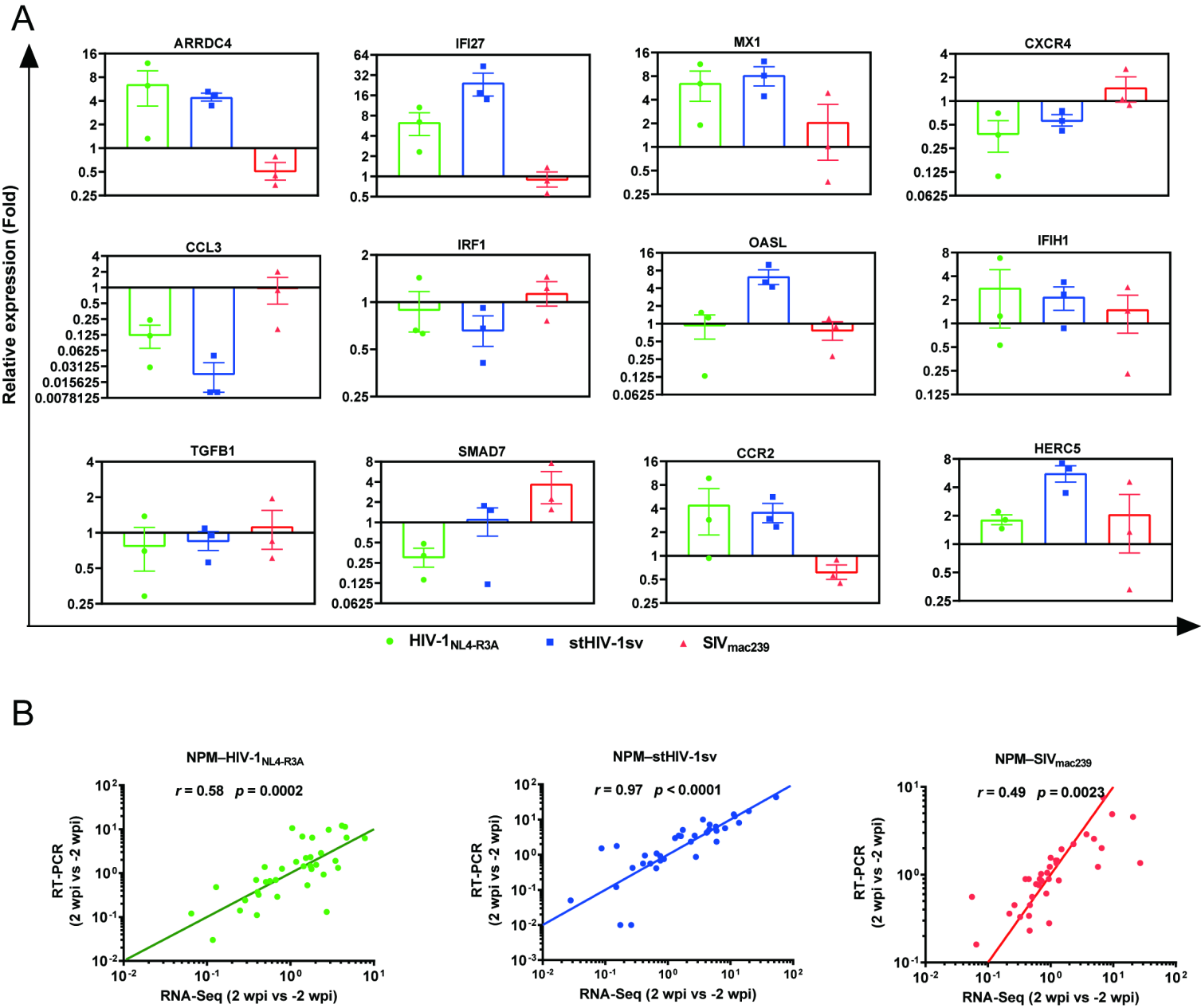


**Fig. S5. Confirmation by qRT-PCR of some differentially expressed genes (DEGs) that have been revealed by global transcriptional analysis in HIV-1_NL4-R3A_-, stHIV-1sv- and SIV_mac239_- infected northern pig-tailed macaques (NPMs).** The expression levels of 12 genes (2 wpi /-2 wpi) were determined by a relative quantitative real-time PCR assay (**A**). Correlations between relative fold changes were measured by transcriptional analysis (RNA-Seq) and qRT-PCR were determined by the Pearson test for HIV-1_NL4-R3A_ (**B**), stHIV-1sv (**C**) and SIV_mac239_ (**D**) infections in NPMs.

**
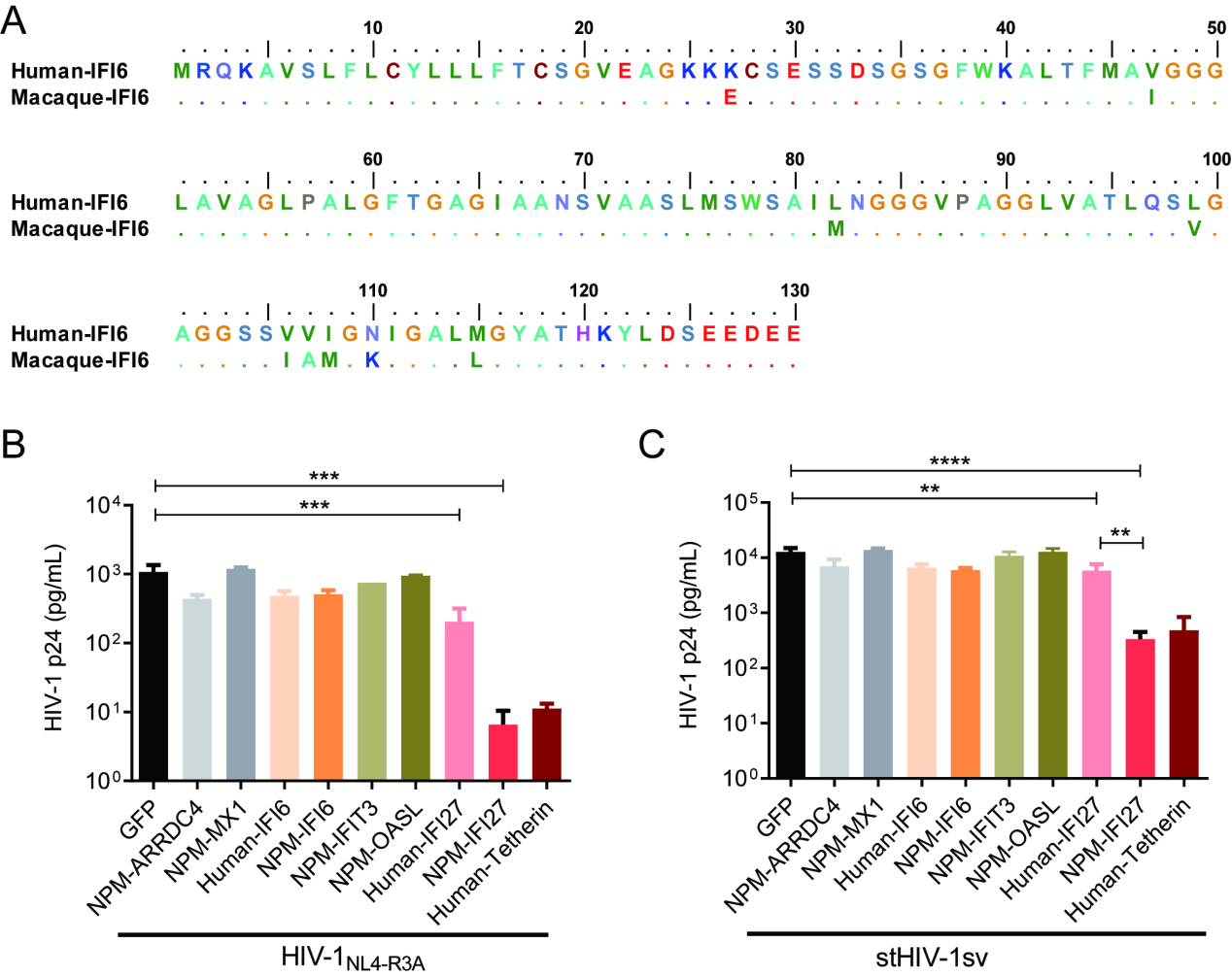
**

**Fig. S6. Anti-pHIV-1_NL4-R3A_ and pstHIV-1sv packaging activities in 293T cells from IFI6, IFI27, and MX1.** (**A**) Sanger sequencing showed protein sequences of IFI6 in NPMs and RMs were identical, but displayed multiple specific amino-acid substitutions compared to that of human. (**B**, **C**) NPM IFI27 shows stronger anti-pHIV-1_NL4.3-R3A_ (**B**) and pstHIV-1sv (**C**) packaging activities than ARRDC4, MX1, IFI6, IFIT3, and OASL in NPMs, as well as IFN27 and IFI6 in humans (one-way ANOVA test between each group. ** *p*<0.01, *** *p*<0.001, **** *p*<0.0001).

**Table S1. *Macaca leonina* genome sequencing statistics.**

| **Pair-end libraries** | **Insert size** | **Total data (G)** | **Read length (bp)** | **Sequence coverage (X)** |
| --- | --- | --- | --- | --- |
| Illumina reads | 250 bp | 105.00 | 150 | 35.00 |
|  | 450 bp | 97.12 |  | 32.37 |
|  | 2 kb | 30.44 |  | 10.15 |
|  | 5 kb | 52.69 |  | 17.56 |
|  | 10 kb | 68.77 |  | 22.92 |
|  | 15 kb | 71.36 |  | 23.79 |
| Total | - | 425.38 | - | 141.79 |

**Table S2. Statistics of the genome assembly for *Macaca leonina*.**

| **Sample ID** | **Length** | | **Number** | |
| --- | --- | --- | --- | --- |
|  | **Contig^**^ (bp)** | **Scaffold (bp)** | **Contig^**^** | **Scaffold** |
| Total | 2,847,382,549 | 2,882,301,780 | 275,818 | 177,242 |
| Max | 404,168 | 20,192,224 | - | - |
| Length>=2kb | - | - | 93,885 | 5,466 |
| N50 | 50,058 | 5,199,335 | 16,913 | 158 |
| N60 | 40,026 | 4,029,715 | 23,270 | 221 |
| N70 | 30,843 | 3,051,171 | 31,356 | 303 |
| N80 | 22,247 | 2,056,069 | 42,173 | 417 |
| N90 | 13,114 | 1,022,076 | 58,571 | 611 |

^**^Contig after scaffolding.

**Table S3. Statistics of the gene prediction of *Macaca leonina* using three annotation strategies.**

| **Geneset** | | **Number** | **CDS + intron length (bp)** | **Average CDS length (bp)** | **Average exons per gene** | **Average exon length (bp)** | **Average intron length (bp)** |
| --- | --- | --- | --- | --- | --- | --- | --- |
| De novo | Augustus | 34,109 | 19,965.28 | 1,014.88 | 5.22 | 194.34 | 4,488.37 |
|  | GlimmerHMM | 594,779 | 4,130.44 | 384.02 | 2.36 | 162.65 | 2,752.50 |
|  | SNAP | 93,568 | 41,456.07 | 585.22 | 4.45 | 131.48 | 11,843.16 |
|  | Genscan | 53,999 | 36,304.48 | 1,059.96 | 6.48 | 163.54 | 6,430.04 |
|  | Geneid | 98,714 | 13,010.99 | 411.88 | 2.70 | 152.51 | 7,408.36 |
| Homolog | *Homo sapiens* | 32,740 | 10,151.45 | 634.96 | 3.39 | 187.17 | 3,977.86 |
|  | *Mus musculus* | 27,967 | 20,604.71 | 1,270.34 | 6.13 | 207.14 | 3,766.91 |
|  | *Pan troglodytes* | 37,623 | 7,528.14 | 536.56 | 2.83 | 189.68 | 3823.09 |
|  | *Pongo abelii* | 15,567 | 11,058.56 | 701.69 | 3.73 | 188.35 | 3799.88 |
|  | *Gorilla gorilla* | 21,842 | 9,625.21 | 632.47 | 3.31 | 191.26 | 3898.30 |
|  | *Rhinopithecus roxellana* | 21,986 | 11,375.97 | 821.47 | 3.65 | 225.21 | 3,986.59 |
|  | *Macaca mulatta* | 31,870 | 121,704.66 | 818.85 | 3.66 | 223.61 | 45,413.22 |
|  | *Macaca nemestrina* | 46,437 | 8,998.83 | 593.99 | 3.22 | 184.32 | 3,781.57 |
| RNA-seq | Cufflinks | 63,170 | 24,937.11 | 2,641.34 | 5.77 | 457.72 | 4,673.48 |
|  | PASA | 94,717 | 20,163.49 | 1,028.15 | 6.18 | 166.43 | 3,695.65 |
| EVM | | 37,865 | 20,050.73 | 973.35 | 5.26 | 185.18 | 4,482.31 |
| PASA-update | | 37,432 | 21,834.54 | 1,007.97 | 5.44 | 185.43 | 4,695.02 |
| Final set | | 23,570 | 29,636.87 | 1,279.35 | 7.17 | 178.48 | 4,597.36 |

**Table S4. Evaluation of the assembly completeness for the *Macaca leonina*.** C: Complete Single-Copy BUSCOs. D: Complete Duplicated BUSCOs. F: Fragmented BUSCOs. M: Missing BUSCOs. N: Total BUSCO groups searched.

| **Species name** | **Genome size** | **BUSCO assessment** |
| --- | --- | --- |
| *Macaca leonina* | 2,882 Mbp | C: 89%, D: 5.6 %, F: 4.7%, M: 5.6%, n: 843 |

**Table S5. Positively selected genes of the *Macaca leonina* lineage.** The *p* values with ≤0.05 are decided by χ^2^ test.

| **Assembly Gene ID** | **Ensembl Gene ID** | **Symbol** | **2*abs(Δℓ)** | ***p*** |
| --- | --- | --- | --- | --- |
| evm.model.scaffold4063.35 | ENSMMUG00000019900 | GNAO1 | 91.07 | 1.39E-21 |
| evm.model.scaffold24799.66 | ENSMMUG00000015918 | KCNQ2 | 39.01 | 4.23E-10 |
| evm.model.scaffold13897.18 | ENSMMUG00000002610 | MARCO | 39.01 | 4.231E-10 |
| evm.model.scaffold945.1 | ENSMMUG00000045491 | NCKAP5 | 37.64 | 8.53E-10 |
| evm.model.scaffold21471.1 | ENSMMUG00000021839 | GRID2IP | 35.96 | 2.01E-09 |
| evm.model.scaffold17255.29 | ENSMMUG00000005401 | FAM189A2 | 35.30 | 2.83E-09 |
| evm.model.scaffold21149.15 | ENSMMUG00000018222 | RHOT1 | 32.52 | 1.18E-08 |
| evm.model.scaffold23821.30 | ENSMMUG00000005898 | MORC2 | 32.25 | 1.35E-08 |
| evm.model.scaffold15205.16 | ENSMMUG00000005597 | KHSRP | 30.51 | 3.32E-08 |
| evm.model.scaffold3073.6 | ENSMMUG00000014292 | OTOF | 29.59 | 5.35E-08 |
| evm.model.scaffold10031.7 | ENSMMUG00000021924 | NHSL2 | 26.06 | 3.31E-07 |
| evm.model.scaffold7001.20 | ENSMMUG00000003130 |  | 24.84 | 6.24E-07 |
| evm.model.scaffold16825.59 | ENSMMUG00000006529 | ASIC1 | 24.69 | 6.75E-07 |
| evm.model.scaffold2659.16 | ENSMMUG00000015985 | KDM4C | 24.65 | 6.86E-07 |
| evm.model.scaffold10165.40 | ENSMMUG00000015601 | SLAMF6 | 23.67 | 1.14E-06 |
| evm.model.scaffold24485.304 | ENSMMUG00000007785 | BACE1 | 22.72 | 1.87E-06 |
| evm.model.scaffold22685.107 | ENSMMUG00000010680 | SEMA4G | 22.44 | 2.17E-06 |
| evm.model.scaffold24791.573 | ENSMMUG00000002692 | TKFC | 22.26 | 2.38E-06 |
| evm.model.scaffold8073.100 | ENSMMUG00000014137 | ADGRL1 | 22.09 | 2.60E-06 |
| evm.model.scaffold19341.11 | ENSMMUG00000045276 | MUC22 | 21.67 | 3.24E-06 |
| evm.model.scaffold8651.82 | ENSMMUG00000016316 | TESK1 | 21.49 | 3.56E-06 |
| evm.model.scaffold3003.35 | ENSMMUG00000005986 | KLF17 | 21.12 | 4.32E-06 |
| evm.model.scaffold16245.193 | ENSMMUG00000017242 | MYCBPAP | 21.06 | 4.44E-06 |
| evm.model.scaffold24969.28 | ENSMMUG00000030902 | NXPE2 | 21.06 | 4.45E-06 |
| evm.model.scaffold9761.3.1 | ENSMMUG00000020726 | DPYD | 20.68 | 5.42E-06 |
| evm.model.scaffold23595.86 | ENSMMUG00000004933 | METTL6 | 20.48 | 6.01E-06 |
| evm.model.scaffold22983.45 | ENSMMUG00000021638 | OR52A5 | 19.46 | 1.03E-05 |
| evm.model.scaffold12415.137 | ENSMMUG00000001008 | SYNE2 | 19.45 | 1.03E-05 |
| evm.model.scaffold7521.10 | ENSMMUG00000007974 | QRFPR | 19.43 | 1.04E-05 |
| evm.model.scaffold1665.178 | ENSMMUG00000015599 | RNASE11 | 19.40 | 1.06E-05 |
| evm.model.scaffold13791.11 | ENSMMUG00000022840 | ZAN | 19.33 | 1.10E-05 |
| evm.model.scaffold4109.38 | ENSMMUG00000012374 | MGAT4B | 19.12 | 1.23E-05 |
| evm.model.scaffold25203.22 | ENSMMUG00000016220 | TRIT1 | 18.90 | 1.38E-05 |
| evm.model.scaffold7161.76 | ENSMMUG00000015395 | PDCD5 | 18.80 | 1.45E-05 |
| evm.model.scaffold24859.34.1 | ENSMMUG00000000751 | NLE1 | 18.61 | 1.61E-05 |
| evm.model.scaffold6059.41 | ENSMMUG00000004742 | ZIC1 | 18.43 | 1.76E-05 |
| evm.model.scaffold969.28 | ENSMMUG00000022562 | IL1RAP | 17.69 | 2.60E-05 |
| evm.model.scaffold6697.33 | ENSMMUG00000022841 | UTP4 | 17.20 | 3.36E-05 |
| evm.model.scaffold25233.44 | ENSMMUG00000011914 | PROX1 | 16.78 | 4.21E-05 |
| evm.model.scaffold10285.55 | ENSMMUG00000021724 | NUP214 | 16.71 | 4.36E-05 |
| evm.model.scaffold19351.2 | ENSMMUG00000023194 | FAM221A | 16.61 | 4.59E-05 |
| evm.model.scaffold24855.5 | ENSMMUG00000007197 | BRCA2 | 16.55 | 4.74E-05 |
| evm.model.scaffold17077.9 | ENSMMUG00000017098 |  | 16.32 | 5.34E-05 |
| evm.model.scaffold14613.14 | ENSMMUG00000017761 | ZNF518B | 15.75 | 7.22E-05 |
| evm.model.scaffold8501.192 | ENSMMUG00000003987 | FHAD1 | 15.63 | 7.71E-05 |
| evm.model.scaffold24249.16 | ENSMMUG00000018344 | MYO15A | 15.62 | 7.74E-05 |
| evm.model.scaffold18203.5 | ENSMMUG00000020064 | F9 | 15.30 | 9.17E-05 |
| evm.model.scaffold23329.11 | ENSMMUG00000014831 |  | 15.20 | 9.68E-05 |
| evm.model.scaffold15733.97 | ENSMMUG00000048466 | PGC | 15.01 | 1.07E-04 |
| evm.model.scaffold15835.1 | ENSMMUG00000004903 | ENPP2 | 14.41 | 1.47E-04 |
| evm.model.scaffold89.66 | ENSMMUG00000004039 | OXER1 | 14.35 | 1.52E-04 |
| evm.model.scaffold5459.2 | ENSMMUG00000001618 | ATP6V0D2 | 14.16 | 1.68E-04 |
| evm.model.scaffold23273.16.1 | ENSMMUG00000003368 | PLXNB3 | 14.08 | 1.75E-04 |
| evm.model.scaffold23595.11 | ENSMMUG00000009636 | CLDN18 | 14.07 | 1.76E-04 |
| evm.model.scaffold7359.2 | ENSMMUG00000009100 |  | 13.50 | 2.39E-04 |
| evm.model.scaffold23059.44 | ENSMMUG00000019873 | CYP4B1 | 13.49 | 2.40E-04 |
| evm.model.scaffold24991.108 | ENSMMUG00000010671 | NOL10 | 13.46 | 2.44E-04 |
| evm.model.scaffold18771.150 | ENSMMUG00000018415 | RASIP1 | 13.41 | 2.51E-04 |
| evm.model.scaffold18771.202 | ENSMMUG00000044817 | NUP62 | 13.35 | 2.58E-04 |
| evm.model.scaffold3083.21 | ENSMMUG00000022441 | SMG1 | 13.24 | 2.75E-04 |
| evm.model.scaffold21547.3 | ENSMMUG00000022244 | HPGDS | 13.22 | 2.78E-04 |
| evm.model.scaffold19171.10 | ENSMMUG00000003526 | POM121L12 | 13.09 | 2.98E-04 |
| evm.model.scaffold3003.18 | ENSMMUG00000012477 | PTPRF | 12.95 | 3.21E-04 |
| evm.model.scaffold17351.44 | ENSMMUG00000029421 | PTPRK | 12.59 | 3.87E-04 |
| evm.model.scaffold6887.83 | ENSMMUG00000006287 | DELE1 | 12.39 | 4.32E-04 |
| evm.model.scaffold13429.6 | ENSMMUG00000004227 | NUMBL | 12.34 | 4.44E-04 |
| evm.model.scaffold4001.101 | ENSMMUG00000015034 | PIP4K2A | 12.26 | 4.64E-04 |
| evm.model.scaffold20857.94 | ENSMMUG00000017114 | PLEKHA6 | 12.12 | 5.00E-04 |
| evm.model.scaffold3751.96 | ENSMMUG00000046155 | TRPM8 | 11.94 | 5.49E-04 |
| evm.model.scaffold21635.37 | ENSMMUG00000004045 | CLUL1 | 11.76 | 6.06E-04 |
| evm.model.scaffold11301.105 | ENSMMUG00000021011 | SPATA7 | 11.65 | 6.40E-04 |
| evm.model.scaffold17851.13 | ENSMMUG00000021660 | AFAP1L1 | 11.55 | 6.76E-04 |
| evm.model.scaffold23523.71 | ENSMMUG00000019209 | NEIL2 | 11.37 | 7.46E-04 |
| evm.model.scaffold23795.12 | ENSMMUG00000011180 | AMN1 | 11.36 | 7.50E-04 |
| evm.model.scaffold1259.30 | ENSMMUG00000000580 | SCN4A | 11.19 | 8.23E-04 |
| evm.model.scaffold22685.147 | ENSMMUG00000020604 | HPS1 | 11.13 | 8.49E-04 |
| evm.model.scaffold21755.19 | ENSMMUG00000042618 | IVL | 11.07 | 8.76E-04 |
| evm.model.scaffold9093.4 | ENSMMUG00000002967 | MOG | 11.06 | 8.82E-04 |
| evm.model.scaffold3003.31 | ENSMMUG00000015158 | SLC6A9 | 11.01 | 9.07E-04 |
| evm.model.scaffold10063.99 | ENSMMUG00000010058 | ATRIP | 10.94 | 9.40E-04 |
| evm.model.scaffold1669.151 | ENSMMUG00000004201 | CHST13 | 10.94 | 9.41E-04 |
| evm.model.scaffold19285.415 | ENSMMUG00000002697 | PICK1 | 10.92 | 9.50E-04 |
| evm.model.scaffold12815.10 | ENSMMUG00000020046 | ALG6 | 10.65 | 1.10E-03 |
| evm.model.scaffold3491.5 | ENSMMUG00000017916 | LIPE | 10.51 | 1.19E-03 |
| evm.model.scaffold14729.16 | ENSMMUG00000018319 | TRIP11 | 10.38 | 1.27E-03 |
| evm.model.scaffold13429.11 | ENSMMUG00000017708 | C19H19orf54 | 10.20 | 1.40E-03 |
| evm.model.scaffold9995.24 | ENSMMUG00000018322 | FCN3 | 10.18 | 1.42E-03 |
| evm.model.scaffold4001.60 | ENSMMUG00000016406 | YME1L1 | 10.08 | 1.50E-03 |
| evm.model.scaffold979.38 | ENSMMUG00000001172 |  | 10.08 | 1.50E-03 |
| evm.model.scaffold13279.124 | ENSMMUG00000039520 | C12orf43 | 10.07 | 1.50E-03 |
| evm.model.scaffold10465.94 | ENSMMUG00000012020 | NCOA3 | 9.91 | 1.64E-03 |
| evm.model.scaffold22947.23 | ENSMMUG00000022481 | CYP1A1 | 9.84 | 1.71E-03 |
| evm.model.scaffold24823.58 | ENSMMUG00000002286 | CREBBP | 9.66 | 1.88E-03 |
| evm.model.scaffold17351.11 | ENSMMUG00000014151 | ENPP1 | 9.64 | 1.91E-03 |
| evm.model.scaffold5453.93 | ENSMMUG00000011198 | FAM169A | 9.34 | 2.24E-03 |
| evm.model.scaffold10165.74 | ENSMMUG00000018128 | NR1I3 | 9.24 | 2.37E-03 |
| evm.model.scaffold13973.2.2 | ENSMMUG00000002955 | CLEC1B | 9.10 | 2.56E-03 |
| evm.model.scaffold16245.98 | ENSMMUG00000019760 | KPNB1 | 9.08 | 2.59E-03 |
| evm.model.scaffold11439.31 | ENSMMUG00000046969 | FCRL3 | 9.04 | 2.64E-03 |
| evm.model.scaffold4001.51 | ENSMMUG00000046971 | RAB18 | 9.03 | 2.66E-03 |
| evm.model.scaffold10903.58 | ENSMMUG00000009325 | KLHDC10 | 8.84 | 2.94E-03 |
| evm.model.scaffold5651.55.2 | ENSMMUG00000022454 | TAX1BP1 | 8.73 | 3.14E-03 |
| evm.model.scaffold24859.13 | ENSMMUG00000022229 | MMP28 | 8.72 | 3.15E-03 |
| evm.model.scaffold2785.81 | ENSMMUG00000049084 | IFT52 | 8.66 | 3.25E-03 |
| evm.model.scaffold2393.11 | ENSMMUG00000008495 | AGBL1 | 8.59 | 3.37E-03 |
| evm.model.scaffold320005.1 | ENSMMUG00000015969 | RSAD2 | 8.49 | 3.57E-03 |
| evm.model.scaffold1129.70 | ENSMMUG00000018070 | NCAPG | 8.36 | 3.84E-03 |
| evm.model.scaffold11275.13 | ENSMMUG00000019751 | PPFIBP2 | 8.26 | 4.04E-03 |
| evm.model.scaffold6821.213 | ENSMMUG00000000934 | STKLD1 | 8.24 | 4.09E-03 |
| evm.model.scaffold16069.4 | ENSMMUG00000000300 | ITGA4 | 8.20 | 4.19E-03 |
| evm.model.scaffold23821.9.4 | ENSMMUG00000020746 | PRR14L | 8.13 | 4.35E-03 |
| evm.model.scaffold5961.4 | ENSMMUG00000009699 | SSUH2 | 8.10 | 4.43E-03 |
| evm.model.scaffold11911.10 | ENSMMUG00000014255 | TLR8 | 8.08 | 4.47E-03 |
| evm.model.scaffold23655.16 | ENSMMUG00000004446 | GGCX | 8.06 | 4.53E-03 |
| evm.model.scaffold24791.593 | ENSMMUG00000000019 | CCDC86 | 8.00 | 4.69E-03 |
| evm.model.scaffold10103.19 | ENSMMUG00000043152 |  | 7.98 | 4.73E-03 |
| evm.model.scaffold7959.2 | ENSMMUG00000009760 | ATP13A5 | 7.96 | 4.78E-03 |
| evm.model.scaffold821.155 | ENSMMUG00000043458 | TMEM178A | 7.91 | 4.93E-03 |
| evm.model.scaffold18975.7 | ENSMMUG00000009668 | TMCO5A | 7.87 | 5.03E-03 |
| evm.model.scaffold1027.251 | ENSMMUG00000029062 | SPIRE2 | 7.84 | 5.10E-03 |
| evm.model.scaffold19915.24 | ENSMMUG00000008540 | RNF112 | 7.80 | 5.24E-03 |
| evm.model.scaffold1743.19 | ENSMMUG00000018510 | SCN9A | 7.68 | 5.58E-03 |
| evm.model.scaffold995.80 | ENSMMUG00000010306 | CLCA4 | 7.64 | 5.71E-03 |
| evm.model.scaffold12299.32.1 | ENSMMUG00000008137 | PIP5K1C | 7.57 | 5.92E-03 |
| evm.model.scaffold14585.13 | ENSMMUG00000015890 | C7H15orf40 | 7.56 | 5.96E-03 |
| evm.model.scaffold7691.93 | ENSMMUG00000002645 | DDX56 | 7.53 | 6.07E-03 |
| evm.model.scaffold18087.108 | ENSMMUG00000001244 | EPB41L5 | 7.50 | 6.17E-03 |
| evm.model.scaffold22059.2 | ENSMMUG00000020565 | CBR1 | 7.38 | 6.60E-03 |
| evm.model.scaffold24477.43 | ENSMMUG00000012969 | PSAP | 7.37 | 6.63E-03 |
| evm.model.scaffold17667.12 | ENSMMUG00000005362 | ADAT2 | 7.22 | 7.20E-03 |
| evm.model.scaffold4081.10 | ENSMMUG00000014891 | CA2 | 7.16 | 7.46E-03 |
| evm.model.scaffold17351.15 | ENSMMUG00000017549 | ENPP3 | 7.10 | 7.70E-03 |
| evm.model.scaffold1637.55 | ENSMMUG00000016695 | CD320 | 7.10 | 7.71E-03 |
| evm.model.scaffold15835.5 | ENSMMUG00000002595 | COL14A1 | 7.09 | 7.75E-03 |
| evm.model.scaffold23403.11.1 | ENSMMUG00000009887 | MLX | 7.04 | 7.96E-03 |
| evm.model.scaffold10677.17 | ENSMMUG00000008738 | COMMD10 | 6.95 | 8.39E-03 |
| evm.model.scaffold24785.82 | ENSMMUG00000000388 | PLEC | 6.79 | 9.17E-03 |
| evm.model.scaffold7161.171 | ENSMMUG00000000845 | ATP4A | 6.77 | 9.26E-03 |
| evm.model.scaffold8937.19.3 | ENSMMUG00000006348 | DENND1A | 6.71 | 9.61E-03 |
| evm.model.scaffold13791.27 | ENSMMUG00000041566 | TSC22D4 | 6.62 | 1.01E-02 |
| evm.model.scaffold2021.6 | ENSMMUG00000023478 | C1orf115 | 6.59 | 1.03E-02 |
| evm.model.scaffold9661.75 | ENSMMUG00000017222 | DGKQ | 6.58 | 1.03E-02 |
| evm.model.scaffold15237.4 | ENSMMUG00000021072 | CEP162 | 6.57 | 1.04E-02 |
| evm.model.scaffold22637.15 | ENSMMUG00000001290 | ASB13 | 6.55 | 1.05E-02 |
| evm.model.scaffold3003.30 | ENSMMUG00000015156 | CCDC24 | 6.53 | 1.06E-02 |
| evm.model.scaffold23595.42 | ENSMMUG00000021694 | AMOTL2 | 6.49 | 1.08E-02 |
| evm.model.scaffold18215.80 | ENSMMUG00000020277 | DOC2A | 6.42 | 1.13E-02 |
| evm.model.scaffold20857.28 | ENSMMUG00000013602 | CR2 | 6.40 | 1.14E-02 |
| evm.model.scaffold24791.206 | ENSMMUG00000013265 | PPFIA1 | 6.39 | 1.15E-02 |
| evm.model.scaffold7855.96 | ENSMMUG00000013659 | MRPS11 | 6.32 | 1.19E-02 |
| evm.model.scaffold1741.25 | ENSMMUG00000015654 | TRMO | 6.30 | 1.21E-02 |
| evm.model.scaffold12215.66 | ENSMMUG00000022278 | MTERF2 | 6.23 | 1.25E-02 |
| evm.model.scaffold6689.18 | ENSMMUG00000017952 | CD2AP | 6.17 | 1.30E-02 |
| evm.model.scaffold23237.6 | ENSMMUG00000000658 | CDHR1 | 6.15 | 1.32E-02 |
| evm.model.scaffold1743.23 | ENSMMUG00000013251 | XIRP2 | 6.11 | 1.34E-02 |
| evm.model.scaffold23523.34 | ENSMMUG00000043605 | RP1L1 | 5.93 | 1.49E-02 |
| evm.model.scaffold19735.18 | ENSMMUG00000021244 | KLHL40 | 5.93 | 1.49E-02 |
| evm.model.scaffold19775.58 | ENSMMUG00000000390 | CLCC1 | 5.91 | 1.50E-02 |
| evm.model.scaffold20763.4 | ENSMMUG00000016104 | CSPG4 | 5.87 | 1.54E-02 |
| evm.model.scaffold23059.13 | ENSMMUG00000023432 | CCDC17 | 5.85 | 1.56E-02 |
| evm.model.scaffold10127.55 | ENSMMUG00000017150 | MAVS | 5.84 | 1.56E-02 |
| evm.model.scaffold24921.8 | ENSMMUG00000045093 | CCNB3 | 5.63 | 1.76E-02 |
| evm.model.scaffold11413.15 | ENSMMUG00000015685 | ZNF804B | 5.63 | 1.77E-02 |
| evm.model.scaffold18215.56 | ENSMMUG00000029159 | ZNF48 | 5.56 | 1.84E-02 |
| evm.model.scaffold24015.31 | ENSMMUG00000006359 | PACSIN3 | 5.53 | 1.87E-02 |
| evm.model.scaffold22227.36 | ENSMMUG00000019237 | ECI1 | 5.44 | 1.97E-02 |
| evm.model.scaffold25001.14 | ENSMMUG00000009365 | SPDL1 | 5.33 | 2.10E-02 |
| evm.model.scaffold4109.69 | ENSMMUG00000017587 | GRM6 | 5.33 | 2.10E-02 |
| evm.model.scaffold3921.88 | ENSMMUG00000015658 | IGF2R | 5.32 | 2.10E-02 |
| evm.model.scaffold5859.26 | ENSMMUG00000004060 | MAPK8 | 5.31 | 2.12E-02 |
| evm.model.scaffold8497.226 | ENSMMUG00000022324 | FTCD | 5.30 | 2.14E-02 |
| evm.model.scaffold19181.3 | ENSMMUG00000003199 | CD200R1 | 5.23 | 2.22E-02 |
| evm.model.scaffold20713.95 | ENSMMUG00000018976 | ADGRV1 | 5.22 | 2.23E-02 |
| evm.model.scaffold24791.558.5 | ENSMMUG00000006655 | MYRF | 5.07 | 2.44E-02 |
| evm.model.scaffold6401.134.2 | ENSMMUG00000004287 | DAXX | 5.01 | 2.53E-02 |
| evm.model.scaffold23455.26 | ENSMMUG00000008420 | RSPO1 | 4.90 | 2.69E-02 |
| evm.model.scaffold16585.9 | ENSMMUG00000032465 | CFP | 4.81 | 2.83E-02 |
| evm.model.scaffold299.92 | ENSMMUG00000005916 | SLC6A11 | 4.76 | 2.91E-02 |
| evm.model.scaffold1027.21 | ENSMMUG00000029070 | SLC38A8 | 4.72 | 2.98E-02 |
| evm.model.scaffold1117.4 | ENSMMUG00000003742 | BCHE | 4.72 | 2.98E-02 |
| evm.model.scaffold15573.1 | ENSMMUG00000008399 | SV2B | 4.69 | 3.03E-02 |
| evm.model.scaffold18433.7 | ENSMMUG00000003309 | OVGP1 | 4.63 | 3.14E-02 |
| evm.model.scaffold24791.624 | ENSMMUG00000045775 | MPEG1 | 4.59 | 3.22E-02 |
| evm.model.scaffold5555.91 | ENSMMUG00000032284 | LRRC47 | 4.58 | 3.24E-02 |
| evm.model.scaffold24799.70 | ENSMMUG00000015916 | CHRNA4 | 4.56 | 3.27E-02 |
| evm.model.scaffold7161.136 | ENSMMUG00000001538 | GRAMD1A | 4.56 | 3.27E-02 |
| evm.model.scaffold89.43 | ENSMMUG00000019841 |  | 4.56 | 3.28E-02 |
| evm.model.scaffold23795.30 | ENSMMUG00000015813 | OVCH1 | 4.44 | 3.52E-02 |
| evm.model.scaffold20517.6 | ENSMMUG00000004766 | MINPP1 | 4.41 | 3.58E-02 |
| evm.model.scaffold16007.24 | ENSMMUG00000002223 | SH2B3 | 4.26 | 3.91E-02 |
| evm.model.scaffold8697.16 | ENSMMUG00000005244 | SPRTN | 4.18 | 4.08E-02 |
| evm.model.scaffold319147.1 | ENSMMUG00000015286 | FAM171A1 | 4.10 | 4.28E-02 |
| evm.model.scaffold5453.84 | ENSMMUG00000017897 | ARHGEF28 | 4.10 | 4.29E-02 |
| evm.model.scaffold3715.39 | ENSMMUG00000012889 | PCYT2 | 4.02 | 4.51E-02 |
| evm.model.scaffold6821.73 | ENSMMUG00000005042 | RABL6 | 3.93 | 4.75E-02 |
| evm.model.scaffold4961.57 | ENSMMUG00000004435 | TPCN1 | 3.86 | 4.94E-02 |
| evm.model.scaffold4961.60 | ENSMMUG00000021795 | DDX54 | 3.85 | 4.99E-02 |

**Table S6. Gene Ontology (GO) enrichment analyses of positively selected genes in the *Macaca leonina* lineage involved in immune-related functional categories.** The corrected *p* values with ≤0.05 were decided by Benjamini-Hochberg FDR.

| **Corr. *p*-value** | **Term ID** | **GO taxon** | **GO Name** | **Gene names** |
| --- | --- | --- | --- | --- |
| 1.79E-02 | GO:0031349 | BP | positive regulation of defense response | TKFC, TLR8, SLAMF6, RSAD2, MAVS, PGC |
| 4.65E-02 | GO:0050778 | BP | positive regulation of immune response | TKFC, TLR8, SLAMF6, RSAD2, MAVS, FCN3, PGC |
| 3.42E-02 | GO:0045088 | BP | regulation of innate immune response | TKFC, TLR8, SLAMF6, RSAD2, MAVS |
| 2.10E-02 | GO:0045089 | BP | positive regulation of innate immune response | TKFC, TLR8, SLAMF6, RSAD2, MAVS |
| 4.09E-02 | GO:0002218 | BP | activation of innate immune response | TKFC, TLR8, RSAD2, MAVS |
| 3.36E-02 | GO:0002758 | BP | innate immune response-activating signal transduction | TKFC, TLR8, RSAD2, MAVS |
| 4.83E-02 | GO:0032607 | BP | interferon-alpha production | TLR8, MAVS |
| 4.83E-02 | GO:0032647 | BP | regulation of interferon-alpha production | TLR8, MAVS |
| 4.61E-02 | GO:0032727 | BP | positive regulation of interferon-alpha production | TLR8, MAVS |
| 6.05E-03 | GO:0002376 | BP | immune system process | ITGA4, SH2B3, TKFC, CLEC1B, ENPP2, BRCA2, CLDN18, CR2, ENPP1, TLR8, PIP4K2A, SLAMF6, CHRNA4, RSAD2, CD320, MAVS, ENPP3, FCN3, MMP28, TMEM178A, PGC |
| 4.61E-02 | GO:0009615 | BP | response to virus | TKFC, TLR8, RSAD2, MAVS, FCN3 |
| 4.83E-02 | GO:0001819 | BP | positive regulation of cytokine production | TKFC, TLR8, SLAMF6, RSAD2, MAVS, IL1, RAP |
| 4.61E-02 | GO:0032755 | BP | positive regulation of interleukin-6 production | TLR8, MAVS, IL1, RAP |
| 3.52E-02 | GO:1903900 | BP | regulation of viral life cycle | PROX1, RSAD2, MAVS, FCN3 |
| 4.83E-02 | GO:0045069 | BP | regulation of viral genome replication | PROX1, RSAD2, MAVS |
| 1.29E-02 | GO:0048525 | BP | negative regulation of viral process | PROX1, RSAD2, MAVS, FCN3 |
| 8.03E-03 | GO:1903901 | BP | negative regulation of viral life cycle | PROX1, RSAD2, MAVS, FCN3 |
| 1.84E-02 | GO:0051607 | BP | defense response to virus | TKFC, TLR8, RSAD2, MAVS, FCN3 |
| 4.83E-02 | GO:0039528 | BP | cytoplasmic pattern recognition receptor signaling pathway in response to virus | TKFC, MAVS |
| 2.28E-02 | GO:0045071 | BP | negative regulation of viral genome replication | PROX1, RSAD2, MAVS |
| 8.40E-03 | GO:0072604 | BP | interleukin-6 secretion | TLR8, MAVS, IL1, RAP |
| 3.10E-03 | GO:2000778 | BP | positive regulation of interleukin-6 secretion | TLR8, MAVS, IL1, RAP |

**Table S7. Positively selected sites (amino-acid sites) of immune-related positively selected genes in the *Macaca leonina* lineage.** The *p* values with ≤0.05 are decided by χ^2^ test.

| **Assembly Gene ID** | **Ensembl Gene ID** | **Gene** | **Positively selected sites** |
| --- | --- | --- | --- |
| evm.model.scaffold11911.10 | ENSMMUG00000014255 | TLR8 | G17A, F27Y, G102R, K674N, L729F, M736T, H737C, S751Y, C762R |
| evm.model.scaffold20857.28 | ENSMMUG00000013602 | CR2 | T385I, M427, M518V, A538K, T552M, T628I, I655T, K847T |
| evm.model.scaffold23595.11 | ENSMMUG00000009636 | CLDN18 | C3I, M11Q, D27N, P47G, I51L |
| evm.model.scaffold17351.15 | ENSMMUG00000017549 | ENPP3 | S209Y, M236V, P337L |
| evm.model.scaffold24799.70 | ENSMMUG00000015916 | CHRNA4 | A301P, G329A |
| evm.model.scaffold15733.97 | ENSMMUG00000048466 | PGC | T78S, S79D |
| evm.model.scaffold10127.55 | ENSMMUG00000017150 | MAVS | M478R |
| evm.model.scaffold13973.2.2 | ENSMMUG00000002955 | CLEC1B | M222R |
| evm.model.scaffold16069.4 | ENSMMUG00000000300 | ITGA4 | S108D |

**Table S8. List of the differentially expressed genes (DEGs) which were enriched in the down-regulated “inflammatory response” pathway following HIV-1_NL4-R3A_, stHIV-1sv, and SIV_mac239_ infections in northern pig-tailed macaques.**

| **Virus** | **Infection**  **stage** | **Adjusted *p* value (-log10)** | **Enriched genes** |
| --- | --- | --- | --- |
| **HIV-1_NL4-R3A_** | Acute infection | 8.85 | CXCL6, CCL7, EPHA2, SBNO2, CD28, CCR4, ICAM1, IL10, CCR6, FPR1, CCL2, FPR2, IL8, FNDC4, PDE2A, FFAR2, CCL3, IL6, CCL4, PTGES, CXCL2, CXCL5, IL1B |
|  | Chronic infection | 15.62 | CCL7, CXCL6, CXCL5, PDE2A, FPR1, CD6, TNIP2, SBNO2, RORA, KDM6B, CCL3, LYN, CCR6, EDNRB, TLR4, SPHK1, IRAK2, LACC1, CCR4, EPHA2, GGT1, WNT5A, CD28, CCR1, NR1D2, ICAM1, TREM1, CXCR4, IL21, HGF, FFAR2, IL10, CXCL2, OLR1, TLR8, IL6, TGM2, PER1, FNDC4, IL1B, CCL20, IL8, CCL4, PTGES |
| **stHIV-1sv** | Acute infection | 9.80 | CXCL2, IL10, SPHK1, EDNRB, CCR6, XCL1, PDE2A, CCL3, CCR4, KDM6B, APOE, IRAK2, ICAM1, TGM2, CCL2, EPHA2, PTGES, FFAR2, IL8, OLR1, FNDC4, IL6, CCL20, CCL4, IL1B, CXCL5 |
|  | Chronic infection | 10.38 | GGT5, CCL7, CCL2, CCL20, FNDC4, CXCL2, RORA, ICAM1, SPHK1, CD28, CD6, CCR6, CCR4, TREM1, APOE, CXCR4, SBNO2, TGM2, IRAK2, KDM6B, FFAR2, EPHA2, PDE2A, PER1, IL10, EDNRB, IL6, IL8, CCL3, CCL4, IL1B, PTGES |
| **SIV_mac239_** | Acute infection | 4.83 | IL1B, IL8, CXCL2, FPR2, TREM1, TREM2, CCL4, EPHA2, CCL3, CCL2 |
|  | Chronic infection | 6.01 | CDK19, CCL4, IL8, CXCL5, TREM1, SBNO2, EPHA2, CXCL2, CCL2 |

**Table S9. List of the differentially expressed genes (DEGs) which were enriched in the up-regulated type I interferon signaling pathway in acute infection of HIV-1_NL4-R3A_, stHIV-1sv, and SIV_mac239_.**

| **Virus** | **Adjusted *p* value (-log10)** | **Enriched genes** |
| --- | --- | --- |
| **HIV-1_NL4-R3A_** | 1.37 | MX2, IFI27, MX1 |
| **stHIV-1sv** | 7.83 | IFI27, RSAD2, IFI6, MX1, IFIT2, OASL, MX2, XAF1, IFIT3 |
| **SIV_mac239_** |  | None |

**Table S10. Information and quality control parameters for transcriptional data (Picard Tools software, http://broadinstitute.github.io/picard/).**

| **Virus** | | **NPM**  **ID** | | **WPI** | | **PCT mRNA bases** | | **PCT mRNA bases (Z-score)** | | **PCT intergenic bases** | | **PCT intergenic bases**  **(Z-score)** | | **Median**  **5 prime to**  **3 prime bias** | | **Median**  **5 prime to**  **3 prime bias (Z-score)** | |
| --- | --- | --- | --- | --- | --- | --- | --- | --- | --- | --- | --- | --- | --- | --- | --- | --- | --- |
| HIV-1_NL4-R3A_ | | 001# | | -2 | | 0.54 | | 0.4 | | 0.31 | | -0.29 | | 0.79 | | -0.56 | |
|  | |  |  | 1 | | 0.51 | | -0.23 | | 0.31 | | -0.28 | | 0.77 | | -0.63 | |
|  | |  |  | 2 | | 0.51 | | -0.15 | | 0.32 | | -0.11 | | 0.84 | | -0.38 | |
|  | |  |  | 3 | | 0.53 | | 0.24 | | 0.32 | | -0.05 | | 0.77 | | -0.61 | |
|  | |  |  | 4 | | 0.5 | | -0.29 | | 0.31 | | -0.22 | | 0.86 | | -0.28 | |
|  | |  |  | 5 | | 0.54 | | 0.31 | | 0.3 | | -0.34 | | 0.85 | | -0.31 | |
|  | |  |  | 6 | | 0.56 | | 0.73 | | 0.29 | | -0.69 | | 1.45 | | 1.93 | |
|  | |  |  | 8 | | 0.44 | | -1.36 | | 0.38 | | 1.12 | | 0.53 | | -1.52 | |
|  | |  |  | 12 | | 0.54 | | 0.37 | | 0.32 | | 0.07 | | 0.8 | | -0.5 | |
|  | |  |  | 18 | | 0.53 | | 0.26 | | 0.34 | | 0.34 | | 0.71 | | -0.87 | |
|  | |  |  | 24 | | 0.55 | | 0.57 | | 0.3 | | -0.32 | | 0.85 | | -0.33 | |
|  | | 002# | | -2 | | 0.54 | | 0.35 | | 0.32 | | -0.08 | | 0.82 | | -0.45 | |
|  | |  |  | 1 | | 0.51 | | -0.1 | | 0.32 | | -0.1 | | 0.81 | | -0.47 | |
|  | |  |  | 2 | | 0.54 | | 0.47 | | 0.32 | | -0.03 | | 0.86 | | -0.28 | |
|  | |  |  | 3 | | 0.55 | | 0.52 | | 0.32 | | -0.09 | | 0.88 | | -0.23 | |
|  | |  |  | 4 | | 0.53 | | 0.21 | | 0.31 | | -0.29 | | 0.82 | | -0.44 | |
|  | |  |  | 5 | | 0.52 | | 0.05 | | 0.31 | | -0.2 | | 0.82 | | -0.42 | |
|  | |  |  | 6 | | 0.42 | | -1.8 | | 0.37 | | 0.92 | | 0.65 | | -1.06 | |
|  | |  |  | 8 | | 0.55 | | 0.66 | | 0.31 | | -0.17 | | 0.85 | | -0.31 | |
|  | |  |  | 12 | | 0.51 | | -0.19 | | 0.33 | | 0.08 | | 0.84 | | -0.37 | |
|  | |  |  | 18 | | 0.51 | | -0.07 | | 0.33 | | 0.12 | | 0.79 | | -0.54 | |
|  | |  |  | 24 | | 0.54 | | 0.48 | | 0.31 | | -0.18 | | 0.85 | | -0.32 | |
|  | | 003# | | -2 | | 0.41 | | -1.99 | | 0.42 | | 1.93 | | 0.98 | | 0.17 | |
|  | |  |  | 1 | | 0.53 | | 0.21 | | 0.31 | | -0.27 | | 0.84 | | -0.38 | |
|  | |  |  | 2 | | 0.56 | | 0.81 | | 0.28 | | -0.86 | | 1.37 | | 1.62 | |
|  | |  |  | 3 | | 0.56 | | 0.79 | | 0.3 | | -0.45 | | 0.93 | | -0.03 | |
|  | |  |  | 4 | | 0.56 | | 0.83 | | 0.28 | | -0.75 | | 1.49 | | 2.07 | |
|  | |  |  | 5 | | 0.59 | | 1.38 | | 0.27 | | -1.01 | | 1.51 | | 2.14 | |
|  | |  |  | 6 | | 0.59 | | 1.24 | | 0.28 | | -0.88 | | 1.5 | | 2.1 | |
|  | |  |  | 12 | | 0.52 | | -0.02 | | 0.33 | | 0.17 | | 0.83 | | -0.42 | |
|  | |  |  | 18 | | 0.58 | | 1.08 | | 0.28 | | -0.75 | | 1.53 | | 2.23 | |
|  | |  |  | 24 | | 0.56 | | 0.67 | | 0.3 | | -0.41 | | 0.86 | | -0.29 | |
| stHIV-1sv | | 004# | | -2 | | 0.5 | | -0.25 | | 0.31 | | -0.22 | | 0.75 | | -0.7 | |
|  | |  |  | 1 | | 0.48 | | -0.76 | | 0.31 | | -0.27 | | 0.77 | | -0.64 | |
|  | |  |  | 2 | | 0.51 | | -0.21 | | 0.32 | | -0.1 | | 0.79 | | -0.57 | |
|  | |  |  | 3 | | 0.49 | | -0.53 | | 0.31 | | -0.16 | | 0.78 | | -0.58 | |
|  | |  |  | 4 | | 0.57 | | 0.87 | | 0.27 | | -0.94 | | 1.36 | | 1.6 | |
|  | |  |  | 5 | | 0.52 | | 0.02 | | 0.31 | | -0.15 | | 0.86 | | -0.29 | |
|  | |  |  | 6 | | 0.52 | | 0.08 | | 0.32 | | -0.06 | | 0.83 | | -0.39 | |
|  | |  |  | 12 | | 0.5 | | -0.38 | | 0.32 | | -0.06 | | 0.77 | | -0.62 | |
|  | |  |  | 18 | | 0.51 | | -0.13 | | 0.35 | | 0.49 | | 0.75 | | -0.7 | |
|  | |  |  | 24 | | 0.53 | | 0.22 | | 0.32 | | -0.09 | | 0.8 | | -0.53 | |
|  | | 005# | | -2 | | 0.53 | | 0.15 | | 0.3 | | -0.37 | | 0.9 | | -0.13 | |
|  | |  |  | 1 | | 0.55 | | 0.55 | | 0.31 | | -0.22 | | 0.83 | | -0.4 | |
|  | |  |  | 2 | | 0.53 | | 0.28 | | 0.31 | | -0.23 | | 0.83 | | -0.4 | |
|  | |  |  | 3 | | 0.54 | | 0.47 | | 0.31 | | -0.17 | | 0.85 | | -0.31 | |
|  | |  |  | 4 | | 0.53 | | 0.18 | | 0.31 | | -0.13 | | 0.82 | | -0.44 | |
|  | |  |  | 5 | | 0.55 | | 0.64 | | 0.31 | | -0.28 | | 0.87 | | -0.26 | |
|  | |  |  | 8 | | 0.55 | | 0.64 | | 0.31 | | -0.14 | | 0.91 | | -0.09 | |
|  | |  |  | 12 | | 0.48 | | -0.76 | | 0.32 | | 0.05 | | 0.78 | | -0.59 | |
|  | |  |  | 18 | | 0.56 | | 0.71 | | 0.29 | | -0.62 | | 1.04 | | 0.38 | |
|  | |  |  | 24 | | 0.54 | | 0.42 | | 0.3 | | -0.34 | | 0.85 | | -0.33 | |
|  | | 006# | | -2 | | 0.48 | | -0.63 | | 0.34 | | 0.43 | | 0.7 | | -0.87 | |
|  | |  |  | 1 | | 0.5 | | -0.4 | | 0.31 | | -0.23 | | 0.8 | | -0.51 | |
|  | |  |  | 2 | | 0.52 | | -0.02 | | 0.32 | | 0.02 | | 0.87 | | -0.27 | |
|  | |  |  | 3 | | 0.56 | | 0.79 | | 0.29 | | -0.67 | | 1.3 | | 1.35 | |
|  | |  |  | 4 | | 0.54 | | 0.4 | | 0.29 | | -0.53 | | 1.28 | | 1.27 | |
|  | |  |  | 5 | | 0.54 | | 0.41 | | 0.29 | | -0.53 | | 1.38 | | 1.64 | |
|  | |  |  | 6 | | 0.54 | | 0.41 | | 0.32 | | -0.07 | | 0.85 | | -0.32 | |
|  | |  |  | 12 | | 0.55 | | 0.51 | | 0.3 | | -0.42 | | 0.98 | | 0.17 | |
|  | |  |  | 18 | | 0.58 | | 1.13 | | 0.29 | | -0.64 | | 1.49 | | 2.05 | |
|  | |  |  | 24 | | 0.55 | | 0.65 | | 0.3 | | -0.32 | | 0.91 | | -0.11 | |
| SIV_mac239_ | | 007# | | -2 | | 0.53 | | 0.21 | | 0.31 | | -0.19 | | 0.83 | | -0.42 | |
|  | |  |  | 1 | | 0.55 | | 0.54 | | 0.31 | | -0.18 | | 0.81 | | -0.49 | |
|  | |  |  | 2 | | 0.53 | | 0.3 | | 0.31 | | -0.26 | | 0.73 | | -0.78 | |
|  | |  |  | 3 | | 0.53 | | 0.18 | | 0.3 | | -0.33 | | 0.82 | | -0.45 | |
|  | |  |  | 4 | | 0.49 | | -0.46 | | 0.31 | | -0.26 | | 0.78 | | -0.6 | |
|  | |  |  | 5 | | 0.51 | | -0.15 | | 0.31 | | -0.16 | | 0.78 | | -0.61 | |
|  | |  |  | 6 | | 0.56 | | 0.85 | | 0.21 | | -2.11 | | 1.5 | | 2.09 | |
|  | |  |  | 8 | | 0.53 | | 0.17 | | 0.32 | | -0.11 | | 0.81 | | -0.47 | |
|  | |  |  | 12 | | 0.53 | | 0.28 | | 0.32 | | -0.01 | | 0.79 | | -0.55 | |
|  | |  |  | 18 | | 0.54 | | 0.46 | | 0.32 | | 0.04 | | 0.73 | | -0.78 | |
|  | |  |  | 24 | | 0.54 | | 0.46 | | 0.32 | | -0.1 | | 0.74 | | -0.73 | |
|  | | 008# | | -2 | | 0.54 | | 0.41 | | 0.3 | | -0.49 | | 1.35 | | 1.53 | |
|  | |  |  | 1 | | 0.54 | | 0.3 | | 0.32 | | -0.03 | | 0.83 | | -0.39 | |
|  | |  |  | 2 | | 0.53 | | 0.13 | | 0.29 | | -0.67 | | 1.36 | | 1.58 | |
|  | |  |  | 3 | | 0.53 | | 0.17 | | 0.3 | | -0.42 | | 1.4 | | 1.72 | |
|  | |  |  | 4 | | 0.55 | | 0.62 | | 0.29 | | -0.62 | | 1.36 | | 1.57 | |
|  | |  |  | 5 | | 0.53 | | 0.25 | | 0.29 | | -0.59 | | 1.4 | | 1.73 | |
|  | |  |  | 6 | | 0.51 | | -0.24 | | 0.3 | | -0.37 | | 1.3 | | 1.36 | |
|  | |  |  | 8 | | 0.53 | | 0.14 | | 0.34 | | 0.41 | | 0.89 | | -0.18 | |
|  | |  |  | 12 | | 0.54 | | 0.36 | | 0.31 | | -0.16 | | 1.38 | | 1.65 | |
|  | |  |  | 18 | | 0.56 | | 0.75 | | 0.3 | | -0.38 | | 1.36 | | 1.58 | |
|  | |  |  | 24 | | 0.52 | | 0.06 | | 0.34 | | 0.38 | | 0.82 | | -0.43 | |
|  | | 009# | | -2 | | 0.52 | | -0.05 | | 0.33 | | 0.23 | | 0.65 | | -1.07 | |
|  | |  |  | 1 | | 0.53 | | 0.23 | | 0.3 | | -0.36 | | 0.85 | | -0.32 | |
|  | |  |  | 2 | | 0.54 | | 0.35 | | 0.3 | | -0.39 | | 0.84 | | -0.38 | |
|  | |  |  | 3 | | 0.52 | | 0.05 | | 0.29 | | -0.63 | | 1.22 | | 1.04 | |
|  | |  |  | 4 | | 0.55 | | 0.58 | | 0.28 | | -0.81 | | 1.37 | | 1.62 | |
|  | |  |  | 5 | | 0.53 | | 0.21 | | 0.29 | | -0.71 | | 1.38 | | 1.67 | |
|  | |  |  | 6 | | 0.53 | | 0.23 | | 0.3 | | -0.36 | | 1.44 | | 1.87 | |
|  | |  |  | 8 | | 0.49 | | -0.45 | | 0.31 | | -0.26 | | 1.35 | | 1.53 | |
|  | |  |  | 12 | | 0.53 | | 0.29 | | 0.31 | | -0.24 | | 0.79 | | -0.53 | |
|  | |  |  | 18 | | 0.53 | | 0.21 | | 0.32 | | -0.06 | | 0.87 | | -0.26 | |
|  | |  |  | 24 | | 0.53 | | 0.26 | | 0.32 | | -0.12 | | 0.91 | | -0.1 | |

**Table S11. Primers used to verify different expressing genes.**

| **Gene** | **Forward (5' - 3')** | **Reverse (5' - 3')** |
| --- | --- | --- |
| **SMAD7** | GTGGGGAGGCTCTACTGTGTC | GGCTACCGGCTGTTGAAGATG |
| **ARRDC4** | GCAGGAAAGAGTCGCCCG | TAAACTTGTGCGGCAATCCTG |
| **CCL17** | AGGGAGCCATTCCCCTTAGAA | TTGGGGTCCGAACAGATGG |
| **MX1** | CTGTGGGCAATCAACCTCCT | TGGCGATGTCCACGTTACTG |
| **CCL3** | GCAACCGGATCTCAGCAACA | TAGGAAGATGACACCGGGCT |
| **CXCR4** | CGACTTCATCTTTGCCAGCG | GGCAGGATAAGGCCAACCAT |
| **HERC5** | AAGACCCTGTTTGGTGGCTG | AGCTGGTCAAGTGTTCCACC |
| **IRF1** | CAACAAACGTGGACGGGAAG | CCCCTGGGCTGTCAATTTCT |
| **STAT1** | AACATTTTGGGCACGCACAC | ACCCTCATTCGTTCTGGTGC |
| **OASL** | TCGATCCCAACAGCTTCGTC | TGTCACTGTCTTGGATGCCG |
| **PLK1** | GCCTGCAGTACATAGAGCGT | GCACCTGCCTTCAGCAAATG |
| **IFIH1** | GGTTGGACTCGGGAATTCGT | AGCTCAGGGTTCATGTAGCG |
| **CCR2** | TGCTGCAAATGAGTGGGTCT | GGTGACCGTCCTGGCTTTTA |
| **TGFB1** | GGACCCTGCCCCTACATTTG | CCACGTAGTACACGATGGGC |
| **IFI6** | GATGAGCTGGTCTGCGATCAT | AGCAGGGCACCTATTTTACCC |
| **IFI27-macaque** | CTGGCTGTGCCGTAGTCTTG | GAAGAGGTGATTCCCGTCGC |
| **IFI27-human** | GCCTCTGCTCTCACCTCATC | CCATGGCCACAACTCCTCC |
| **RPL13A- macaque** | AAGGTGTTTGACGGCATCCC | CTTCTCCTCCAAGGTGGCTGT |
| **GAPDH-human** | CAAGAAGGTGGTGAAGCAGGC | CATACCAGGAAATGAGCTTGAC |
